# Technological variability during the Early Middle Palaeolithic in Western Europe. Reduction systems and predetermined products at the Bau de l'Aubesier and Payre (South-East France)

**DOI:** 10.1371/journal.pone.0178550

**Published:** 2017-06-07

**Authors:** Leonardo Carmignani, Marie-Hélène Moncel, Paul Fernandes, Lucy Wilson

**Affiliations:** 1 IDQP Phd candidate, IPHES, Institut Català de Paleoecologia Humana i Evolució Social, Tarragona, Spain; 2 Àrea de Prehistòria, Universitat Rovira i Virgili (URV), Tarragona, Spain; 3 Dipartimento di Studi Umanistici, Università degli Studi di Ferrara, Ferrara, Italy; 4 External Member of UMR 7041 ArScAn, Anthropologie des Techniques, des Espaces et des Territoires au Pliocène et Pléistocène (AnTET), Maison de l’Archéologie et de l’Ethnologie, Nanterre, France; 5 UMR7194 – HNHP Department of Prehistory (CNRS – MNHN – UPVD – Sorbonne Universités), Paris, France; 6 Paleotime, Villars-de Lens, France; 7 Department of Biological Sciences, University of New Brunswick in Saint John, Saint John, New Brunswick, Canada; Max Planck Institute for the Science of Human History, GERMANY

## Abstract

The study of the lithic assemblages of two French sites, the Bau de l’Aubesier and Payre, contributes new knowledge of the earliest Neanderthal techno-cultural variability. In this paper we present the results of a detailed technological analysis of Early Middle Palaeolithic lithic assemblages of MIS 8 and 7 age from the two sites, which are located on opposite sides of the Rhône Valley in the south-east of France. The MIS 9–7 period is considered in Europe to be a time of new behaviours, especially concerning lithic strategies. The shift from the Lower Palaeolithic to the Early Middle Palaeolithic is “classically” defined by an increase in the number of core technologies, including standardized ones, which are stabilized in the full Middle Palaeolithic (MIS 5–3), associated with the decline of the “Acheulean” biface. Applying a common technological approach to the analysis of the two assemblages highlights their technological variability with respect to reduction systems and end products. Differences between Payre and the Bau de l’Aubesier concerning raw material procurement and faunal exploitation only partially explain this multifaceted technological variability, which in our opinion also reflects the existence of distinct technological strategies within the same restricted geographic area, which are related to distinct traditions, site uses, and/or as yet unknown parameters.

## Introduction

The MIS 9 to 7 time-span in Europe, roughly 350,000 to 200,000 B.P., is considered to have recorded a behavioural change commonly described as the shift from the Lower to the Middle Palaeolithic or again as the threshold from Mode 2, including bifaces, to Mode 3, linked to the development of different core technologies [1]. From a general point of view the continuity in biface production and the increase in predetermined flaking systems, even if not generalizable, are recurrent features which are valid during all this period on a continental scale. Attribution of an assemblage to the Upper Acheulean (UA) or to the early Middle Palaeolithic (EMP) is often based on the proportion of bifaces and/or pebble tools alongside flake standardized production. This distinction does not take into consideration the mosaic of changes found over time between MIS 9 and 6 at various sites. In fact, it is difficult to find a clear chronological and behavioural boundary between the Lower and Middle Palaeolithic which could be used to name the assemblages of this age.

Over this large chronological timespan, we find not only new technological features, but also other changes regarding subsistence strategies, such as the wooden throwing spears discovered at Schöningen, Germany [2] and recently re-dated to the MIS 9, that provide evidence of specialized hunting [3].

The iconic example of the development of more complex flaking technology is the rise of the Levallois concept, which is based on preparation of the core volume (convexities, striking platform) in order to obtain blanks of a predetermined shape by means of a parallel exploitation of the flaking surface [4]. Early evidence of Levallois technology is most commonly documented in Western Europe at the end of MIS 9 [5, 6, 7, 8, 9, 10, 11, 12, 13, 14, 15, 16, 17, 18, 19], although the emergence of this concept is recognized, sporadically, in a few earlier sites: in France at Cagny la Garenne and Cagny Cemetery dated to MIS 12–11 [20, 21, 22, 23, 24], in the Iberian Peninsula at Grand Dolina TD10 and Ambrona dated to MIS 10–9 [25, 26, 27, 28, 29] and more recently in the Italian peninsula at Guado San Nicola dated to the end of MIS 11-beginning of MIS 10 [30].

**Fig 1:**
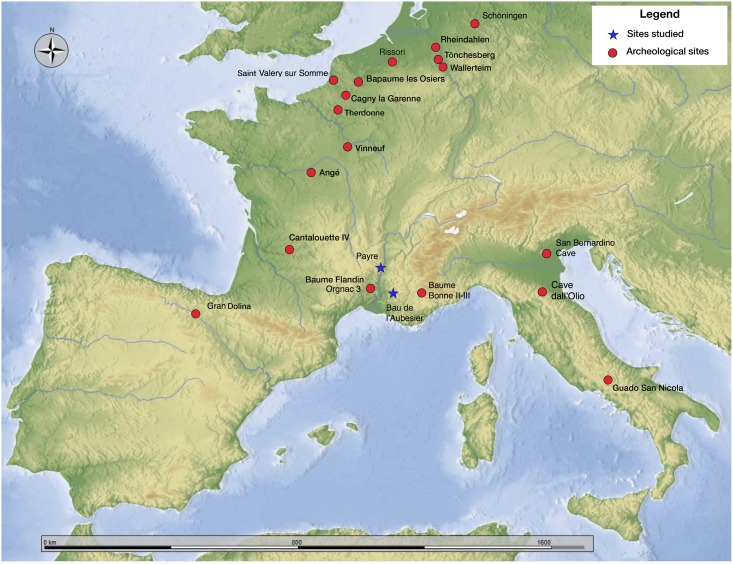
Location of the Bau de l’Aubesier and Payre and of the main sites cited in this paper.

Another element of variability in reduction strategies that partially overlaps the rise of the Levallois concept during the EMP is the development of blade production in northern Europe, either by volumetric systems or by *débitage* of a surface [31, 32]. Early evidence of laminar production dates back to MIS 7 and the end of MIS 8 in the north of Europe, for instance at the sites of Saint-Valéry-sur-Somme [33], Bapaume-les Osiers [34] and Therdonne [35] in France, and Rissori [36, 37] in Belgium. Unlike bifacial and Levallois production, which can be considered as a more global phenomenon, blade production was restricted to northern Europe for a long time. However by MIS 5 blade production covered a larger area, including northeast Germany in the site of Tönchesberg [38], and Wallertheim [39], and in central and southern France, the sites of Angé [40], Vinneuf [41], Baume Flandin [42, 43, 44], Cantalouette [45] and Baume Bonne [46, 47] (Fig 1). In all of these sites blades rarely assumed a dominant role but co-existed with a variety of reduction systems (Levallois, Discoid, etc.) as well as with shaping systems, such as at the sites of Bapaume-les Osiers [34] and Vinneuf [41].

In parallel with these new trends in core technologies, bifaces persisted throughout the EMP and into the late Middle Palaeolithic [48]. In south-western France, the MTA industries record shaping processes as part of the Neanderthal techno-cultural equipment during the late Middle Palaeolithic (MIS 4–3), although their features are not comparable to the Acheulian bifaces [49, 50, 51, 52].

Even from this brief overview it is clear that it is extremely difficult to define a unique trend in lithic technological development that can be valid at a large scale of analysis. Depending on the geographic scale of analysis and the choice of parameters used to describe the lithic industries, different scenarios can be created. The problems connected to the choice of the scale of analysis for the comprehension of material culture in prehistory have been underlined by several authors [see for example [53, 54]. Using as a primary technological parameter the distinction between shaping and flaking processes in assemblages during the EMP, we recognize two variants: (1) industries only due to flaking technologies, and (2) industries where biface and flaking reduction systems co-exist in various proportions. At the European continental scale these two categories are ubiquitous and are not linked to a specific geographic area. On the other hand, if we reduce our scale of analysis by taking into account more of the details of the reduction systems, it is possible to distinguish macro-areas, such as in the case of northern European blade production.

Over the last few years, new approaches in lithic studies, better-defined chronologies and the discovery of new sites have helped us with the recognition of specific technological features. Recently some authors have proposed tracing the onset of some regional differentiation in the technological behavioural changes starting from the Lower Palaeolithic [55, 56, 57]. This complex scenario has generated widespread debate about the definition of the chronological limits between the Lower and the Middle Palaeolithic as well as on the definition of the relevant archaeological data to be considered as the marker of these behavioural changes [58, 12, 13, 59, 60, 61, 62]. If the evidence of technological variability during the Middle Palaeolithic is now commonly accepted, the causes of this variability are still in contention. This question, which originated in the transatlantic debate between Binford [63, 64] and Bordes [65, 66], has continued and is still one of the central topics in the understanding of material culture. Different explanations of the possible causes of technological variability have been proposed in the last decades: climatic change, raw material economy, subsistence strategy, demography, or mobility patterns. To reduce the impact of external factors, the analysis of technological features needs to be tested in a small geographic area with a common environmental context. Furthermore, to identify the particular the technological features used by the human groups, we have to go further than a macro-technological subdivision (i.e., Levallois: Non-Levallois; Biface: Non-Biface) especially if applied on a large geographical scale.

For all these reasons, the main aim of this paper is to discuss the technological changes that affect the EMP through a detailed technological analysis applied on a small regional scale. The assemblages of the Bau de l’Aubesier and Payre, located in south-eastern France on opposite sides of the Rhône corridor, are considered through a detailed comparative technological analysis. The choice of these two sites is motivated by geographical and chronological parameters: (1) the two sites yielded layers dated to the MIS 8 and MIS 7, a crucial period of time for understanding the technological changes to the EMP in Western Europe; (2) they are located within the same region and in similar environments. A basic question guides our analysis: Does technological variability on a regional scale exist in the EMP and if so, is it due to external factors and constraints, or is it evidence of diversification of the techno-cultural traditions of human groups as early as the EMP?

## Materials and methods

### The sites of Payre and the Bau de l’Aubesier

#### Payre

Located in the Rhône Valley (south-eastern France) (Fig 1), Payre is a small cave above the confluence of the Rhône and Payre Rivers at the intersection of various biotopes [67, 68, 69, 70]. The 5m thick stratigraphic sequence yielded 8 occupation layers in 4 phases (units). The basal units G and F that we investigate here are dated by several radiometric methods (ESR, TL, U-Th, TIMS) to MIS 8–7, roughly 250,000 to 200,000 years before present [71, 72]. The biochronology places the bottom of the sequence (units G and F) in a temperate event (beginning of MIS 7). A chronological boundary is visible between the top of unit F and level D, dated to the end of MIS 6. The units are sub-divided into several levels including levels Ga, Gb and levels Fa, Fb, Fc, Fd. Unit G is composed of 6 lenses or sedimentological sub-layers. Level Ga is a dense concentration of artefacts related to lenses G4 and G5, 50 to 65 cm thick and composed of many small blocks. Unit F is composed of 7 lenses or sedimentological sub-layers. Level Fb is strictly related to the grey lens F3, 15–20 cm thick and free of limestone blocks. Unit G was excavated over 50 m^2^ and unit F over 20 m^2^.

Human occupations were identified on the basis of the vertical and horizontal distributions of pieces. The density of pieces at different levels indicates distinct phases of occupation, the remains of palimpsests close in time, as is commonly observed in cave sites. Some horizontal lithic refittings attest to only a slight displacement of material, confirmed by refittings of faunal remains and anatomical connections found *in situ*. Moreover, the thin edges of the artefacts are still relatively fresh, which indicates that post-depositional processes in each unit have been minor. The lithic material found in units G and F is attributed to the Early Middle Palaeolithic, with a standardized discoidal and orthogonal core technology on flint and mainly scrapers and points [57]. Some heavy-duty tools, as well as crudely-made bifaces and pebble tools, were made *in situ* or outside the site on local quartzite, limestone and basalt [57]. New evidence of use wear analysis on quartzite has been recently published [73].

#### The Bau de l’Aubesier

**Fig 2:**
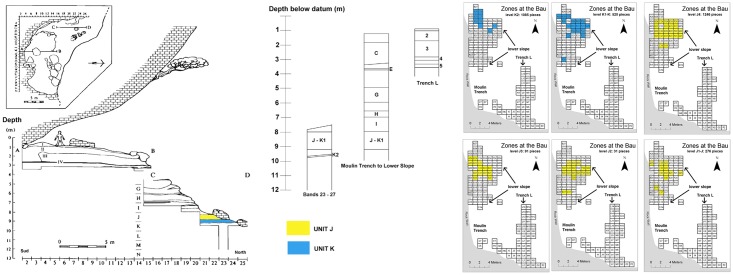
Simplified stratigraphy of the Bau de l’Aubesier. Drawings on the left after Lebel (2000a, p.22). On the left and in the center: simplified stratigraphy. In colour (yellow and blue), Units J and K. On the right the plan distribution of the lithic collection for each sub-unit considered in this study.

The Bau de l’Aubesier is a large rock shelter located in the gorges of the Nesque River, Vaucluse (south-eastern France) (Fig 1). The site, known since the beginning of the 20th century [74, 75], was extensively excavated from 1987 to 2000 by an international team led by Serge Lebel, then of the Université du Québec à Montréal, Canada [76, 77]. The deposits in the site are complex, both laterally and vertically, and include more than 60 different sedimentological layers and lenses over a total thickness of more than 13 metres. The deposits also include at least a dozen archaeological levels, divided into more than 30 sub-layers, according to sedimentological, archaeological, or arbitrary depth criteria (Fig 2). The archaeological levels have been identified based on the density of archaeological pieces within the geological layers, and their vertical distribution, indicating distinct phases of occupation throughout the sequence. Only a few lithic refittings could be made, which may be due to pieces having been carried out of the site. The material, depending on the level, can be highly patinated but is otherwise relatively fresh, without indication of strong crushing or displacement between levels. Based on radiometric, faunal and stratigraphic results, it appears that the entirety of the deposits dates to between roughly 100,000 (or less) and 250,000 years ago [78, 79, 80]. The lower part of the site has been attributed to the later Middle Pleistocene, and the upper part to the Late Pleistocene [81, 82]. The two levels discussed here, J and K, are the lowermost ones. No direct radiometric dates are available for these levels, but based on their position in the stratigraphy, the dating of the layers above them, and their faunal content (discussed below), they can be attributed to MIS 7.

This present study concerns the lowest archaeological layers, J and K, which were divided during the excavation into J, J1, J2, J3 and J4, and K, K1 and K2 respectively. The lowest level, K2, is a layer of fine sediments with some larger rocks, probably reflecting accumulation during a temperate and relatively warm phase during MIS 7. This was followed by cooler phases during which more cryoclastic debris fell from the roof and walls of the rock shelter. During this time period, archaeological layers K1 through J also accumulated. These were later washed, reworked and eroded, forming a shallow basin or gully which later layers filled in and covered over. The total thickness of this phase of the deposits amounts to approximately 120 cm. There are very few traces of fire: only about 3% of remains in layer J and 5% of remains in layer K show any trace of having been burned, and there are no hearths or concentrations of burned material. The densest archaeological accumulations are in layers J4 and K2 (Fig 2). One hominin tooth (an incisor) was found in layer J and has been described as pre-Neanderthal, archaic Homo [81]. All together, layers J and K provided both lithic (almost entirely flint) and faunal remains attesting to significant use of the site by early Middle Palaeolithic hominins.

## Methods

The comparison between the lithic collections uses both qualitative and quantitative parameters, describing the entire assemblages through an extensive technological analysis. A preliminary sorting procedure has been done dividing the lithic collection into two broad categories: undetermined and determined pieces. We classified as determined pieces all the removals that can be linked to specific reduction strategies (e.g. Levallois, Discoid) or methods (e.g. unidirectional, centripetal). Deeply patinated pieces or pieces with disorganized scars which did not allow us to attribute them to a specific reduction strategy or method were classified as undetermined pieces.

The qualitative analysis follows the general principles of the *chaîne opératoire*, based on the identification of the distinct phases of the process [83, 84, 85, 86, 87]. Reconstitution of the *chaîne operatoire* is based on the identification of the percussion technique, methods and concepts that underlie the reduction processes [88, 89]. The percussion techniques were identified according to the criteria derived from experimental studies by Pelegrin [90, 91]. Diacritical analysis was applied to cores and blanks in order to identify the chronological order of the scars distinguishing the preparation phases from the main production phases [92, 93]. Due to the scarcity of refitting in the collections, the reduction sequences are described using the mental refitting method proposed by Pelegrin [94]. The mental refitting method involves a comparison between the technological features of the cores (removals, striking platforms) and blanks (organization of the scars), which allows us to identify the phases and purposes of the *débitage*.

The small number of cores in the assemblages did not allow us to quantify them in terms of ratio. A synthetic quantification of the technological systems through the sequence has been done by creating four groups based on the number of cores present in each layer: absent (0), rare (1–2), present (3–5), and abundant (>5).

Identification of the Levallois concept follows the guidelines set out by Boëda [95, 4]. In terms of Discoid production, we used the definition of Boëda [95, 96], and also took into account broader criteria [97, 98].

**Fig 3:**
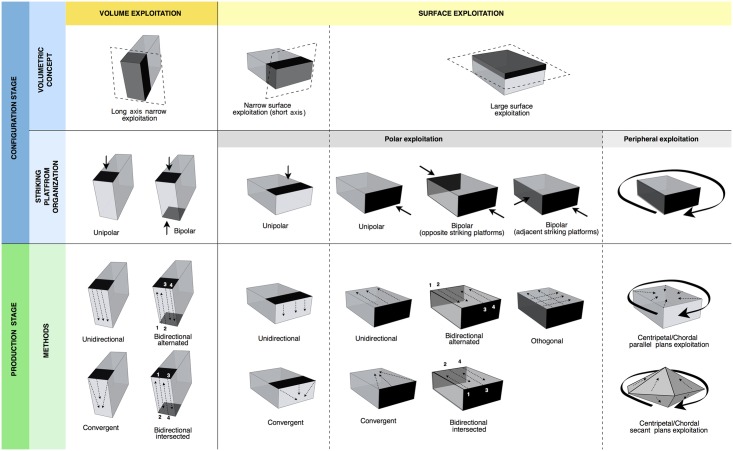
Schematic model of the reduction systems and the terminology used in this paper.

Definition and characterization of the production techniques was preceded by a personal analytical approach which takes into account five technical parameters: the volumetric concept used, the striking platform organisation, the direction and the organization of the removals and the angle between the debitage surface and the striking platform. The combination of these parameters allows us to preliminarily describe and identify the characteristics of the technological systems (Fig 3). Supporting information for the terminology used in this work is provided in S1 File.

The Payre collections total 8275 pieces and the Bau de l’Aubesier total 3249 pieces. The collections of Payre are available at the department of Prehistory, National Museum of Natural History, Institut de Paléontologie Humaine, 1 rue René Panhard, 75013 Paris, France. The collections of Bau de l’Aubseier are stored and available in a building of the French Minsitry of Culture at Vaison la Romaine, France.

The field permits (1990–2002) for Payre were given to M-H. Moncel by the Ministry of Culture, Service Régional de l’Archeologie. The permit (2015–2016) to analyse the lithic collections of Bau de l’Aubesier was given to L. Carmignani and L. Wilson by by the Ministry of Culture, Service Régional de l’Archeologie.

### Composition of the lithic material assemblages

#### Payre

Units G and F yielded 8275 pieces including flakes, cores, pebbles and debris. The main density of pieces is in the sublevels Ga and Fa (Table 1). Small debris (<20mm) are in general abundant and attest to an intense flaking activity *in situ*. Undetermined flakes are also a significant part of the collection (Table 1), due to the huge flaking activity, which produced many broken flakes and debris. The proportion of undetermined flakes larger than 20 mm ranges from 23% in sub-level Fc to 57.9% for sub-level Gb. Determined flake proportions range from 20.4% in sub-level Gb to 2.4% in sub-level Fb. Cores are present in all of the sub levels, ranging from 3.1% of the assemblage in sub-level Gb to 0.2% in sub-level Fb. The few handaxes are crudely made, on large flakes of quartzite or flint flaked outside of the site, or on small flint nodules. The Chi2 value of 27,587 with a df (degree of freedom) = 16, indicates a significant difference in term of composition of the assemblages between the levels (alpha 0,001, 39,252).

**Table 1:** Payre. Composition of the lithic assemblages of units G and F.

Assemblage composition	Gb		Ga		Fd		Fc		Fb		Fa		Total
	n	%	n	%	n	%	n	%	n	%	n	%	n
**Undetermined Flakes<20mm**	122	*20*.*1*	1253	*38*,*2*	284	*51*.*7*	330	*62*.*7*	*577*	*71*.*4*	1477	*59*.*0*	*4043*
**Undetermined Flakes>20mm**	352	*57*.*9*	1176	*35*,*8*	142	*25*.*9*	121	*23*.*0*	*203*	*25*.*1*	667	*26*.*7*	*2661*
**Determined flakes**	89	*14*.*6*	669	*20*,*4*	98	*17*.*9*	44	*8*.*4*	*19*	*2*.*4*	229	*9*.*2*	*1148*
**Cores**	19	*3*.*1*	47	*1*.*4*	17	*3*.*1*	9	*1*.*7*	*2*	*0*.*2*	44	*1*.*8*	*138*
**Entire Pebbles**	16	*2*.*6*	88	*2*.*7*	5	*0*.*9*	11	*2*.*1*	*6*	*0*.*7*	63	*2*.*5*	*189*
**Broken Pebbles**	8	*1*.*3*	49	*1*.*5*	3	*0*.*5*	11	*2*.*1*	*1*	*0*.*1*	21	*0*.*8*	*93*
**Handaxes**	2	*0*.*3*	-	*0*	-	*0*	-	*0*	-	*0*	1	*0*.*04*	*3*
**Total**	608	*100*	3282	*100*	549	*100*	526	*100*	*808*	*100*	2502	*100*	*8275*

Raw materials for flaking are largely dominated by a good quality flint (between 84% and 92%), with quartz or basalt secondary (Table 2). A small quantity of quartzite and limestone was used as well. The raw materials were collected in the form of cobbles, small nodules and flakes.

**Table 2:** Payre. Raw material distribution in units G and F. Flake numbers in brackets represent retouched pieces.

		Flakes<20mm	Flakes>20mm	Cores	Pebbles Entire (Broken)	Handaxe	Total (n.)	Total (%)
**Fa**	Flint	1375	693(269)	40	-	-	2108	*84*.*3*
	Basalt	7	25	-	72(7)	-	104	*4*.*2*
	Quartz	91	121(10)	1	1	-	214	*8*.*6*
	Limestone	4	31	2	5(1)	-	42	*1*.*7*
	Quartzite	-	26(6)	1	6(6)	1	34	*1*.*4*
	**Total**	**1477**	**896**	**44**	**84**	**1**	**2502**	***100***
**Fb**	Flint	549	176(58)	2	-	-	727	90.0
	Basalt	8	17	-	7(1)	-	32	4.0
	Quartz	17	23(1)	-	-	-	40	5.0
	Limestone	-	2	-	-	-	2	*0*.*2*
	Quartzite	3	4	-	-	-	7	*0*.*9*
	**Total**	**577**	**222**	**2**	**7**	-	**808**	***100***
**Fc**	Flint	298	117(50)	9	-	-	424	*80*.*6*
	Basalt	6	15	-	18(2)	-	39	*7*.*4*
	Quartz	26	28(1)	-	1	-	55	*10*.*5*
	Limestone	-	4	-	2(1)	-	6	*1*.*1*
	Quartzite	-	1	-	1	-	2	*0*.*4*
	**Total**	**330**	**165**	**9**	**22**	-	**526**	***100***
**Fd**	Flint	271(1)	213(44)	17	-	-	501	*91*.*3*
	Basalt	3	9	-	8(2)	-	20	*3*.*6*
	Quartz	10	16	-	-	-	26	*4*.*7*
	Limestone	-	-	-	-	-	-	*0*
	Quartzite	-	2(1)	-	-	-	2	*0*.*4*
	**Total**	**284**	**240**	**17**	**8**	-	**549**	***100***
**Ga**	Flint	1253(4)	1482(515)	41	2	-	2778	*84*.*6*
	Basalt	-	173(3)	-	125(38)	-	301	*9*.*2*
	Quartz	-	132(21)	6	2	-	140	*4*.*3*
	Limestone	-	11(2)	-	4	-	15	*0*.*5*
	Quartzite	-	44(15)	-	4(3)	-	48	*1*.*5*
	**Total**	**1253**	**1845**	**47**	**137**	-	**3282**	***100***
**Gb**	Flint	120	422(83)	19	-	2	563	*92*.*6*
	Basalt	-	2	-	23(7)	-	25	*4*.*1*
	Quartz	-	13	-	-	-	13	*2*.*1*
	Limestone	-	1	-	1	-	2	*0*.*3*
	Quartzite	2	3	-	-	-	5	*0*.*8*
	**Total**	**122**	**441**	**19**	**24**	**2**	**608**	***100*.*0***

#### Bau de l’Aubesier

Units K and J yielded 3249 lithic pieces, including cores, flakes and debris. Lithic pieces were mostly concentrated in the sub-levels K2 and J4. Debris <20 cm (undetermined flakes and fragments) are the main part of the collection, residues of an intense flaking activity *in situ*. Some are deeply patinated and thus impossible to determine. Determined flakes are more abundant than at Payre with a frequency ranging from 30.1% in the sub level J-J1 to 7.7% in K2 (Table 3). Cores are rare, between 0.8% in sub-level K-K1 and 4.4% in sub-level J3. A high ratio of cores (16.1%) characterizes the sub-level J2, but this has a total assemblage of only 31 lithic items (Table 3). The Chi2 value of 180,722, with a df = 12, indicates a significant difference in term of composition of the assemblages between the levels (alpha 0,001, 32,909).

**Table 3:** Bau de l’Aubesier. Composition of the lithic assemblages from the lowest part of the sequence.

Assemblage composition	K2		K-K1		J4		J3		J2		J-J1		Total
	n	*%*	n	*%*	n	*%*	n	*%*	n	*%*	n	*%*	n
**Undetermined fragment<20**	670	*61*.*8*	283	*54*.*4*	700	*56*.*2*	28	*30*.*8*	3	*9*.*7*	103	*37*.*3*	1787
**Undetermined fragment>20**	65	*6*.*0*	72	*13*.*8*	191	*15*.*3*	25	*27*.*5*	4	*12*.*9*	55	*19*.*9*	412
**Undetermined flakes<20**	180	*16*.*6*	42	*8*.*1*	97	*7*.*8*	1	*1*.*1*	5	*16*.*1*	19	*6*.*9*	344
**Undetermined flakes>20**	42	*3*.*9*	23	*4*.*4*	51	*4*.*1*	3	*3*.*3*	6	*19*.*4*	10	*3*.*6*	135
**Determined flakes**	84	*7*.*7*	107	*20*.*6*	185	*14*.*8*	30	*33*.*0*	8	*25*.*8*	83	*30*.*1*	514
**Cores**	13	*1*.*2*	4	*0*.*8*	22	*1*.*8*	4	*4*.*4*	5	*16*.*1*	8	*2*.*9*	57
**Total**	1085	*100*	520	*100*	1246	*100*	91	*100*	31	*100*	276	*100*	3249

Flint was used almost exclusively in these levels: the only non-flint piece is a quartzite flake fragment from level J4. A large proportion of these pieces is heavily patinated, and can be identified only as flint. Combined with a very small proportion of flint types of unknown provenance, this means that all together 43.2% of the pieces in levels J-J4 are flint from unknown/unidentifiable sources, as are 51.7% of the pieces from levels K-K2. The sources of the remaining pieces have been identified, and (as will be discussed more fully below) are located within 15 km of the site, along an axis extending towards both the south-west and the north-east.

## Results

### Reduction sequences and the aims of production at Payre

Knapping processes dominate in Units G and F. Shaping processes provide rare bifaces and pebble tools (Table 4). Different schemes of *débitage*, aimed at producing different types of end-products, have been recognized based on the analysis of the cores and determined blanks (Tables 4 and 5).

**Table 4:** Payre. Numbers of the core types throughout the sequence.

Systems structure	Cores techno-type	Gb	Ga	Fd	Fc	Fb	Fa	Tot. Num.	Tot. %
**Peripheral**	**Secant planes cores “Discoid”**	1	2	-	3	-	8	14	10.1
	**Secant planes cores “Partial exploitation”**	7	9	1	2	-	4	23	16.7
	**Secant planes “Trifacial cores”**	3	5	-	-	-	-	8	5.8
	**Parallel planes exploitation**	-	10	2	2	-	8	22	15.9
**Polar**	**Unidirectional parallel planes**	-	3	2	-	-	4	9	6.5
	**Unidirectional “short axis exploitation”**	2	4	-	-	-	2	8	5.8
	**Multidirectional**	3	5	1	2	-	6	17	12.3
	**Bidirectional parallel planes**	-	-	-	-	-	1	1	0.7
	**Orthogonal parallel planes**	-	-	1	-	-	-	1	0.7
	**Convergent parallel planes**	-	1	-	-	-	-	1	0.7
**Volumetric**	**Bladelet cores**	-	-	2	-	-	2	4	2.9
	**Bipolar percussion core**	-	1	-	-	-	-	1	0.7
	**Large flake cores**	1	1	1	-	-	3	6	4.3
	**Undetermined core fragments**	2	6	7	-	2	6	23	16.7
	**TOTAL**	19	47	17	9	2	44	138	100

**Table 5:** Payre. Determined pieces. Numbers in brackets indicate retouched pieces.

Techno-types	Gb		Ga		Fd		Fc		Fb		Fa	
	num	*%*	num	*%*	num	*%*	num	*%*	num	*%*	num	*%*
**Centripetal flakes**	37 (5)	*32*.*5*	337 (83)	*41*.*8*	33 (4)	*31*.*1*	14 (4)	*21*.*2*	12(2)	*46*.*2*	83(22)	*26*.*4*
**Debordant flakes (chordal)**	16 (5)	*14*.*0*	135 (48)	*16*.*7*	12 (1)	*11*.*3*	3	*4*.*5*	-	*0*	37 (4)	*11*.*8*
**Pseudolevallois**	-	*0*	1	*0*.*1*	1	*0*.*9*	1	*1*.*5*	1	*3*.*8*	3	*1*.*0*
**Unipolar flakes**	10 (1)	*8*.*8*	24 (3)	*3*.*0*	13	*12*.*3*	3 (1)	*4*.*5*	3	*11*.*5*	26 (8)	*8*.*3*
**Debordant unipolar flakes**	2	*1*.*8*	5 (1)	*0*.*6*	4	*3*.*8*	-	*0*	-	*0*	1 (1)	*0*.*3*
**Bipolar flakes**	1	*0*.*9*	2	*0*.*2*	-	*0*	2	*3*.*0*	-	*0*	-	*0*
**Debordant bipolar flakes**	-	*0*	1	*0*.*1*	-	*0*	2	*3*.*0*	-	*0*	-	*0*
**Orthogonal flakes**	1	*0*.*9*	5 (3)	*0*.*6*	-	*0*	2	*3*.*0*	-	*0*	-	*0*
**Convergent/sub-convergent flakes**	2 (1)	*1*.*8*	10	*1*.*2*	-	*0*	-	*0*	-	*0*	-	*0*
**Bladelets**	-	*0*	-	*0*	3	*2*.*8*	-	*0*	-	*0*	-	*0*
**Blades**	-	*0*	-	*0*	7	*6*.*6*	-	*0*	-	*0*	-	*0*
**Kombewa**	3	*2*.*6*	27 (5)	*3*.*3*	1	*0*.*9*	1 (1)	*1*.*5*	1	*3*.*8*	19 (1)	*6*.*1*
**Kombewa debordant**	1 (1)	*0*.*9*	4 (2)	*0*.*5*	-	*0*	-	*0*	-	*0*	3	*1*.*0*
**Quina**	3	*2*.*6*	10	*1*.*2*	-	*0*	-	*0*	-	*0*	2	*0*.*6*
**Demi Quina**	2	*1*.*8*	17	*2*.*1*	1	*0*.*9*	-	*0*	-	*0*	6	*1*.*9*
**Wide flake (Demi Quina retouch)**	1	*0*.*9*	1	*0*.*1*	14	*13*.*2*	-	*0*	-	*0*	2	*0*.*6*
**Wide flakes**	-	*0*	21 (7)	*2*.*6*	3 (1)	*2*.*8*	6 (3)	*9*.*1*	1	*3*.*8*	29 (9)	*9*.*2*
**Bifaces**	2	*1*.*8*	-	*0*	-	*0*	-	*0*	-	*0*	1	*0*.*3*
**Macro-tools**	1	*0*.*9*	4	*0*.*5*	1	*0*.*9*	-	*0*	-	*0*	5	*1*.*6*
**Entire Pebble**	16	*14*.*0*	88(41)	*10*.*9*	5(2)	*4*.*7*	11(3)	*16*.*7*	6(1)	*23*.*1*	63(14)	*20*.*1*
**Broken Pebble**	8	*7*.*0*	49	*6*.*1*	3	*2*.*8*	11	*16*.*7*	1	*3*.*8*	21	*6*.*7*
**Striking platform flakes**	2 (1)	*1*.*8*	1	*0*.*1*	-	*0*	7	*10*.*6*	-	*0*	2	*0*.*6*
**Shaping/retouching flakes**	4	*3*.*5*	43	*5*.*3*	1	*0*.*9*	3	*4*.*5*	-	*0*	10	*3*.*2*
**Rejuvenation flakes**	1	*0*.*9*	21 (14)	*2*.*6*	-	*0*	-	*0*	-	*0*	-	*0*
**Crested flakes**	1	*0*.*9*	-	*0*	4	*3*.*8*	-	*0*	1	*3*.*8*	1	*0*.*3*
**Total**	114	*100*	806	*100*	106	*100*	66	*100*	26	*100*	314	*100*

The core technologies are predominantly based on the exploitation of the large surfaces of the volume of the support. Depending on the organization and location of the striking platforms, the flaking follows either a peripheral or a polar management scheme. Marginal volumetric exploitation was used to produce bladelets (Table 4). Centripetal flakes are the most numerous recurrent products in the layers, varying from 46.2% in sub-level Fb to 21.2% in sub-level Fc (Table 5). The second most common category is unipolar flakes. Minor percentages are represented by bipolar, orthogonal, convergent and Kombewa flakes (Table 5).

#### Peripheral exploitation

**Fig 4:**
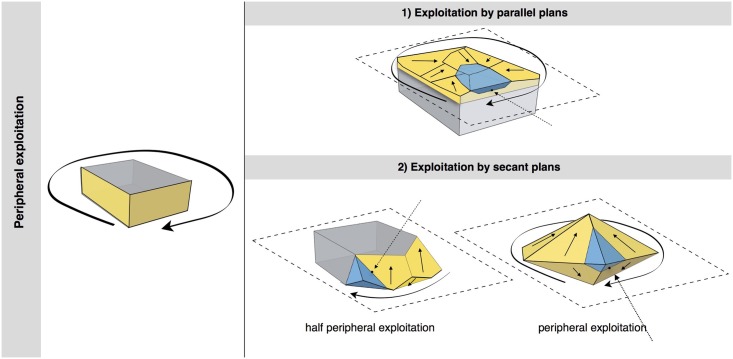
Model of peripheral planes exploitation. On the top right exploitation by parallel planes. On the bottom right the two variants of exploitation by secant planes.

The technological parameters of the flakes fit well with the analyses of the cores. Peripheral exploitation of the core is the main flaking process used at Payre, with an overall proportion of 48.6% (Table 4). This group includes cores with management of the periphery of the volume by either centripetal and/or chordal removals. Based on the detachment angle of the removals, two different cases have been identified: a peripheral secant planes exploitation system and a peripheral parallel planes exploitation system (Fig 4).

**Fig 5:**
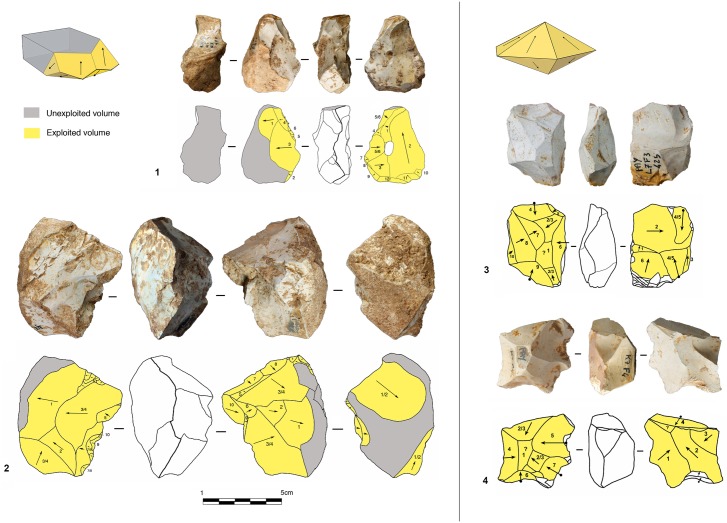
Payre. **Peripheral secant planes cores**. Cores of partial peripheral secant planes exploitation from sub-unit Gb (1, 2). Cores of complete peripheral secant planes exploitation from sub unit Fa (Discoidal) (3,4).

In the secant planes exploitation systems the *débitage* starts without preparation, by a series of secant removals on the two opposite surfaces. The direction of the removal is alternatively centripetal and chordal. Centripetal removals are struck on the center of the flaking surface, producing centripetal flakes with a peripheral cutting edge. Chordal removals give “debordant” flakes, including parts of the core’s edge. Each removal participates in maintaining the convexity and creates a new striking platform for the following removals. In relation to the mode of exploitation, two sub-types have been identified. In the first one, the removals are around the core’s entire periphery (Fig 5, n. 3, 4). This system can be fully ascribed to the classical Discoid systems. In the second type, the removals are limited to one side of the core periphery, leaving the other part of the volume unexploited (Fig 5, n. 1, 2). These two variants are present in both units G and F but in different amounts. Cores with a complete peripheral exploitation (Discoid) increase in abundance in unit F and especially in sub-level Fa (Table 4). Conversely, partial secant exploitation is more frequent in unit G (Table 4).

**Fig 6:**
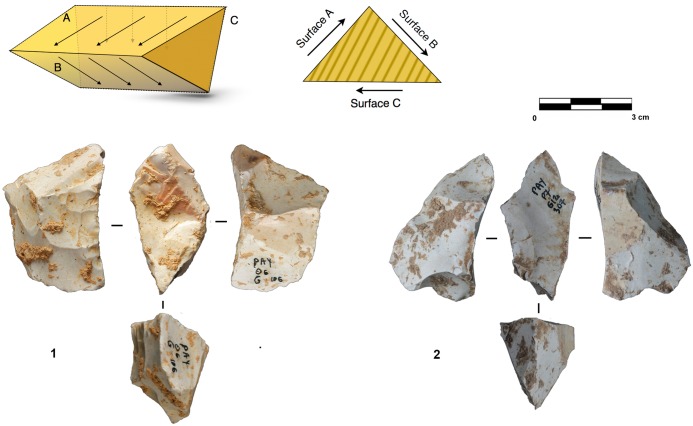
Payre. **Trifacial cores.** Trifacial secant planes exploitation cores from sub-levels Ga (n. 1) and Gb (n.2).

The sub-levels Ga and Gb differ from unit F by having produced 8 cores with a triangular cross-section, here called “Trifacial cores” (Fig 6). The flaking starts with a first series of secant removals without preparation. The second and the third series of removals repeat the same sequence on the two adjacent surfaces using the scars of the first series of removals as a striking platform. The sequence is repeated until the exhaustion of the core.

**Fig 7:**
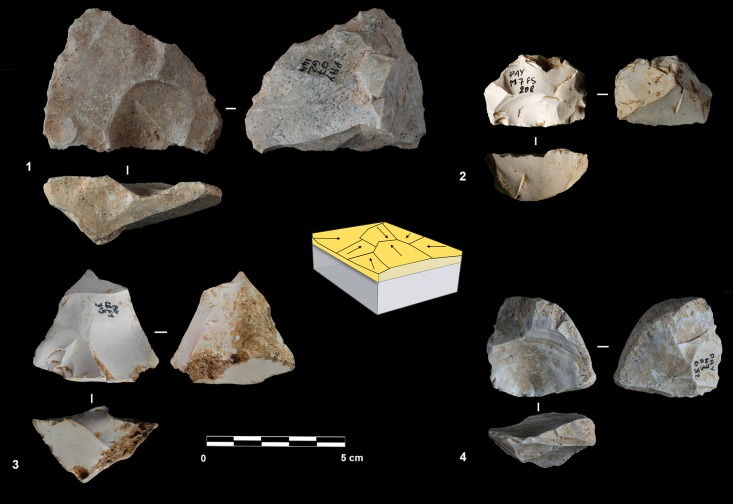
Payre. **Peripheral parallel planes cores.** Cores with peripheral parallel planes exploitation from sub-levels Ga (n. 1 and 2), Fb (n.3) and Fd (n. 4).

Twenty-two cores show a different exploitation. Centripetal and chordal removals are struck from the platforms around the core’s entire periphery but the flaking surface is managed by parallel planes (Fig 7). These cores show some features in common with the definition of Levallois proposed by Boëda [4, 95]. They present asymmetrical convex surfaces (plane of intersection). However, we do not include them in the category of Levallois cores because they lack specific features that characterize this volumetric concept. These cores do not show any scars that would indicate a clear separation between the configuration phase of the *débitage* surface and the main production phase. The striking platforms are minimally prepared. A single centripetal series is obtained on the surface without evidence of preparation of the lateral and distal convexities (Fig 7). After a short series of centripetal removals, the flaking surface is quickly abandoned. No rejuvenation flakes, suggesting a reconfiguration of the core, have been found. These kinds of cores are well represented in both units G (n = 10) and F (n = 12) (Table 4).

**Fig 8:**
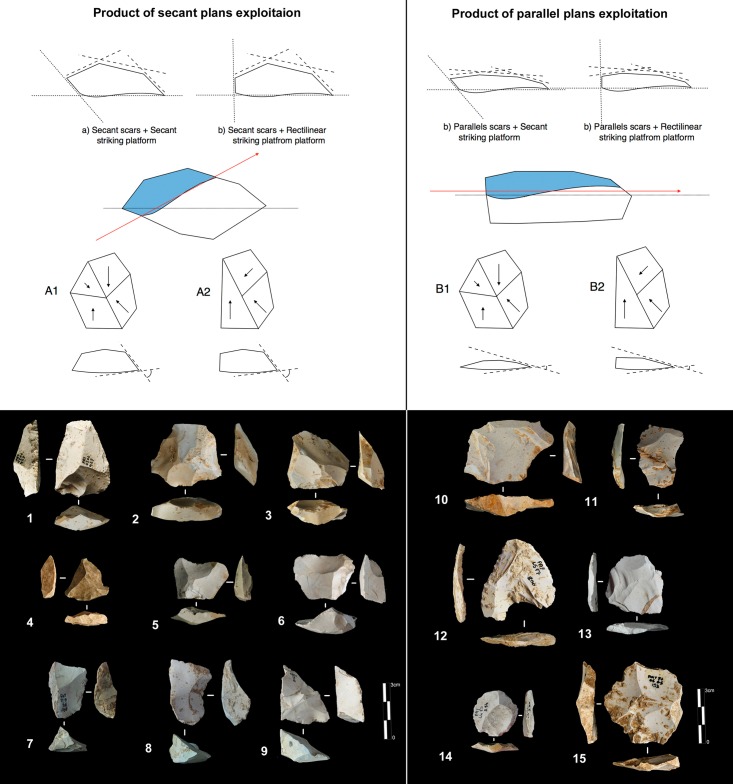
Payre. **Peripheral exploitation blanks.** On the top, sketch of products from a secant planes exploitation (top left) and from a parallel planes exploitation (top right). On the bottom left, blanks of secant planes exploitation: centripetal flakes (type A1) from sub-units Ga (n. 1 to 4), Fd (n. 5), and Fa (n. 6); debordant flakes (type A2) from sub-units Ga (n. 7 to 9). On the bottom right, products of parallel planes exploitation: centripetal flakes (type B1) from sub-units Ga (n. 10 to 13) and Fa (n.14); debordant flakes (type B2) from sub-unit Ga.

Products derived by peripheral exploitation show different features depending on the procedure applied (i.e. parallel or secant plane exploitation). To distinguish between products derived from peripheral secant exploitation and peripheral parallel exploitation, we take into account the angular degree of the dorsal scars and the angular degree between the platform and the ventral surface of the blanks. Secant exploitation produces blanks with an inclined platform and the dorsal surface is characterised by secant centripetal scars (Fig 8). These flakes are short and thick with a robust cutting edge between about 40° and 60°. Centripetal and chordal directions of flaking produce respectively flakes with a peripheral cutting edge (type A1), and debordant flakes (type A2) (Fig 8).

These products are diverse in shape. The cutting edge can be polygonal, sub-circular or convergent.

Flakes with a convergent cutting edge are numerous in both units G and F [99, 100]. Analysis of the dorsal scars rarely shows a convergent method. This result is confirmed by the cores. Just one core in sub-level Ga shows this type of method. Diacritical analysis of the dorsal scars of these convergent pieces shows that they are closer to a peripheral secant planes exploitation technique (Fig 6 n. 4, 9).

Products derived from parallel planes exploitation (Types B1 and B2) (Fig 8) differ greatly from those derived from the previous one. They are close to the typical Levallois flakes (Fig 8 n. 10 to 15). The platform is generally flat, but in some cases is carefully prepared. The angle between the ventral surface and the platform of the flakes is between 95° and 115°. Scars on the dorsal surface are parallel or sub-parallel. Compared with the A1 and A2 flake types, B1 and B2 flake types are thinner, with a cutting edge of about 15° to 40°. Flakes with secant dorsal scars (Type A) are present in all of the sub-levels except Fd (Table A in S2 File). Among the flakes with secant dorsal scars (Type A), the majority are associated with an inclined platform, due to the secant planes exploitation. This is particularly clear in unit F where no rectilinear platform is related to flakes with secant scars (Table B in S2 File).

**Fig 9:**
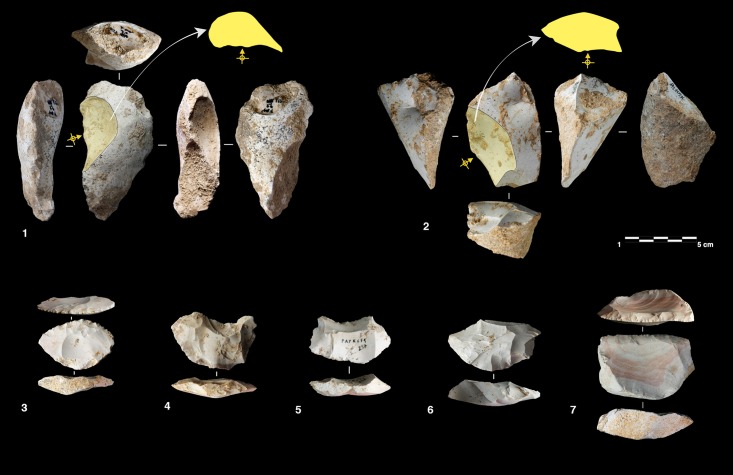
Payre. **Wide flakes production.** Large flake cores (n.1, 2). Retouched wide flakes (n. 3 and 7). Unretouched wide flakes (n. 4 to 6).

Six cores with secant planes were abandoned after a short series of removals. There is no evidence of preparation. Two of these cores come from unit G and three from unit F (Fig 9 n. 1 and 2). These cores can be related to large, wide flakes found in unit G (23 items) and unit F (55 items) with a flat or a cortical platform (Fig 9 n. 3 to 7).

#### Polar exploitation

**Fig 10:**
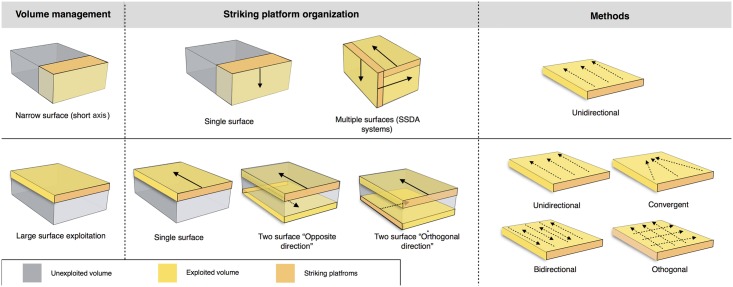
Payre. **Polar exploitation variability.** Model of the polar exploitation variability at Payre.

This system is based on the exploitation of a surface with one or more striking platforms located on one or several sides of the cores. There are 56 such cores in unit F and 18 in unit G (Table 4). Based on the location of the striking platforms on the core, two different types are distinguished. The first is an exploitation of the narrowest surface of the core, while in the second the exploitation is applied to the largest surface of the core (Fig 10).

**Fig 11:**
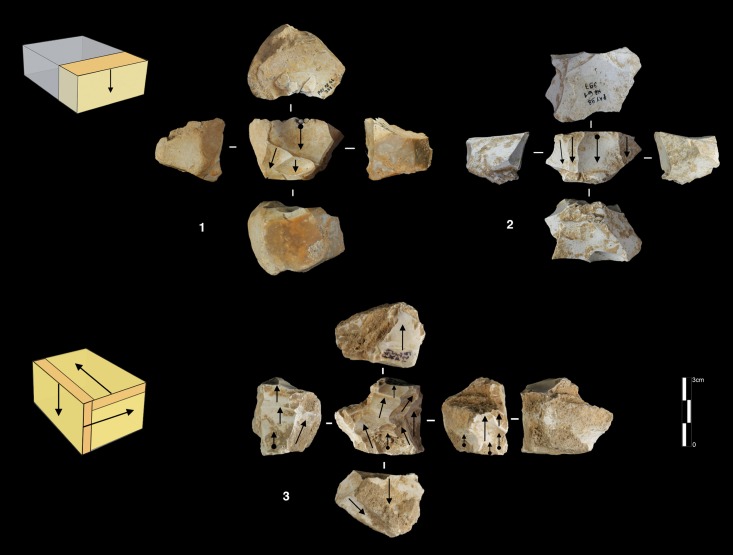
Payre. Unidirectional short axis cores.

The cores managed on the narrowest surface show only unidirectional removals. Six of these cores were found in unit G and two in unit F (Table 4). The removals are directly struck on the core without preparation of the lateral and distal convexities. This does not allow the surface to be exploited for very long: several cores were quickly abandoned after a short series of removals, due to hinged fractures (Fig 11 n. 1). Repetition of a unidirectional series of removals on the same core can give various forms which can be interpreted erroneously as different reduction systems.

A group of 17 cores, 8 found in unit G and 9 in unit F shows exploitation of multiple surfaces (Fig 11 n. 3). The final shape of these cores resembles the SSDA “*systéme de débitage par surface alterné*” cores [101]. In the case of Payre, these cores have to be described as an advanced phase of exploitation by unidirectional series managed on the same volume.

**Fig 12:**
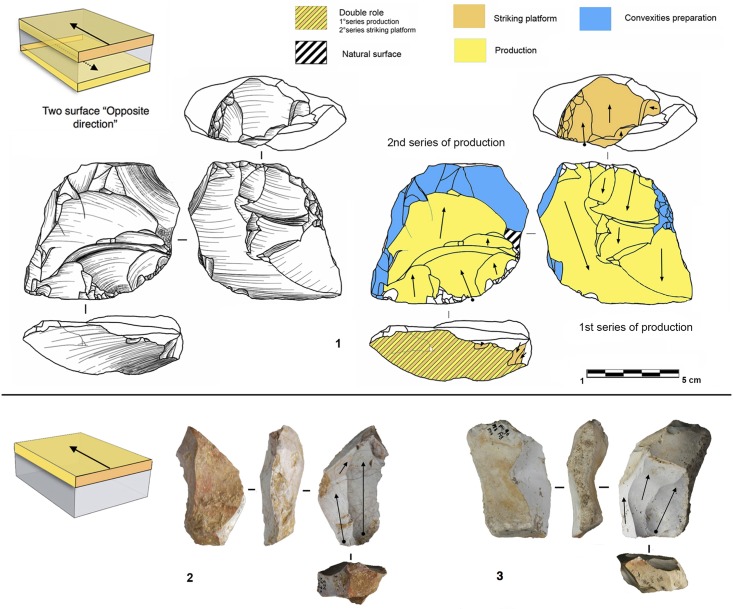
Payre. Unidirectional large surface cores.

The category of cores exploited on the large surface groups together various methods: unidirectional, bidirectional, convergent and orthogonal. The unidirectional method is the most frequent and is equally present in the two units (Table 4). Convergent, orthogonal and bipolar methods are less common. Selection of the appropriate volume allows for exploitation without the preparation of the lateral and distal convexity (Fig 12 n. 2, 3). Just one core shows a partial preparation of the flaking surface (Fig 12 n.1). In this core *débitage* stopped due to hinged fractures and continued on the opposite surface with a second unidirectional series made in the opposite direction to the first (Fig 12 n.1). In other cases, the second series of removals can be made in the same direction as the first one or orthogonally.

**Fig 13:**
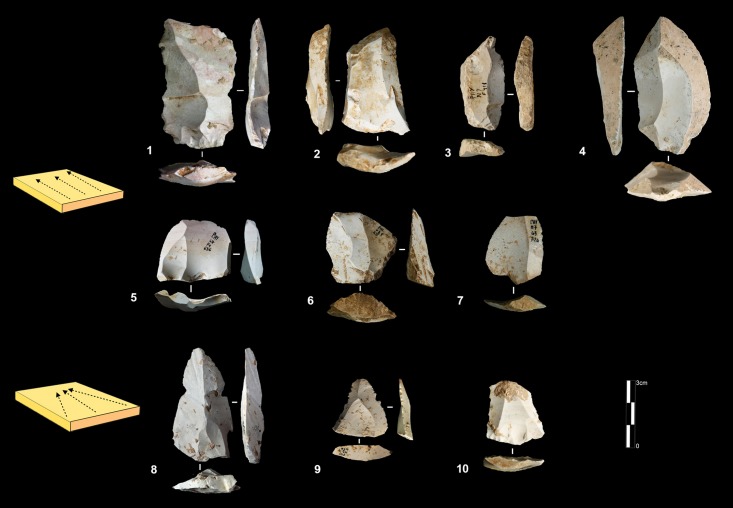
Payre. Elongated unidirectional flakes from Unit F (n. 1 to 3) and Unit G (n.4); Unidirectional short flakes from Unit F (n. 7 to 9) and Unit G (n.10); Convergent flakes from Unit G (n. 8 to 10).

The variability of end-products of polar exploitation is similar to what is observed in the cores. Unidirectional flakes are the most frequent, especially in sub-units Fa and Ga (Table 5). Triangular flakes coming from a convergent method are less frequent, and are more numerous in sub-levels Ga and Gb (Fig 13, n. 8 to 10). Orthogonal and bipolar flakes are as rare as the cores.

Unidirectional methods produce quadrangular slightly elongated flakes with a peripheral cutting edge and debordant flakes (Fig 13, n. 1 to 7). Products from unidirectional exploitation on the narrow surface and unidirectional exploitation on the large surface are similar.

Differences between the unidirectional flakes can however be detected in terms of the elongation. A group of unidirectional flakes shows a tendency to be more elongated and could be related to the exploitation of the largest surfaces (Fig 13, n. 1 to 4). Conversely, the presence of short quadrangular flakes can correspond technologically to the exploitation of the shortest axis (Fig 13, n. 5 to 7).

#### Volumetric exploitation

**Fig 14:**
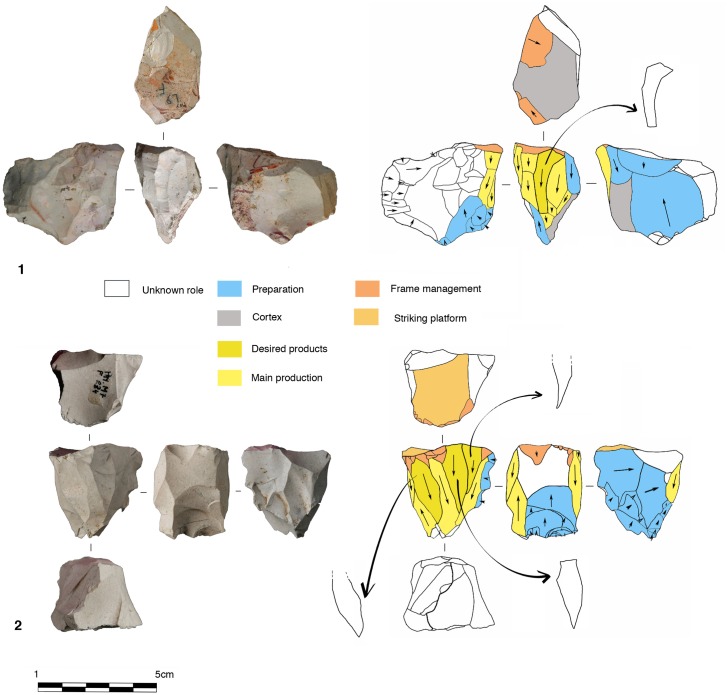
Payre. Bladelet cores from unit F.

Four small cores aimed at the production of bladelets were found in unit F (Table 4). There was minimal preparation of the cores. Partial preparation was made by rear lateral removals aimed at centering the flaking surface (Fig 14). The striking platform was either left cortical or minimally prepared. Only 3 bladelets were found, in sub-level Fd. Despite the lack of these products, the scars on cores clearly indicate production of convergent/sub-convergent bladelets (Fig 14). Export of the products outside of the site is possible, or the core may be a mobile piece since no products or by-products related to this reduction system have been observed in the series.

### Reduction sequences and the aims of production at the Bau de l’Aubesier

The lithic assemblages of units K and J are entirely composed of products derived from flaking systems, with both surface and volumetric management occurring (Table 6). Surface exploitation was recognized on 42 cores and includes both polar and peripheral variants. Volume exploitation is indicated by 14 cores.

**Table 6:** Bau de l’Aubesier. Numbers of core types throughout the sequence.

Systems structure	Core techno-type	K2	K1	K	J4	J3	J2	J1	J	Total
**Peripheral**	Secant planes cores “Discoid”	3	-	-	-	-	-	-	-	3
	Secant planes cores “Partial exploitation”	1	1	-	-	-	-	-	-	2
	Centripetal parallel planes exploitation	1	-	-	2	2	2	-	4	11
**Polar**	Unidirectional parallel planes	-	-	-	1	1	1	1	-	4
	Bidirectional parallel planes	1	-	2	1	-	-	-	-	4
	Convergent parallel planes	1	-	-	1	-	1	-	-	3
	Orthogonal parallel planes	1	-	-	-	1	-	-	-	2
	Multidirectional	-	-	-	5	-	-	1	-	6
**Volumetric**	Convergent semi-rotating	-	-	-	4	-	-	-	2	6
	Unidirectional semi-rotating	1	-	-	4	-	1	-	-	6
	Pyramidal cores	1	-	1	-	-	-	-	-	2
	Large flakes cores	1	-	-	4	-	-	-	-	5
	Undetermined cores fragment	2	-	-	-	-	-	-	-	2
	**Total**	**13**	**1**	**3**	**22**	**4**	**5**	**2**	**6**	56

#### Polar and peripheral parallel planes exploitation systems. Levallois or not Levallois?

Secant plane exploitation is rare at the Bau de l’Aubesier and shows the same variability as at Payre.

**Fig 15:**
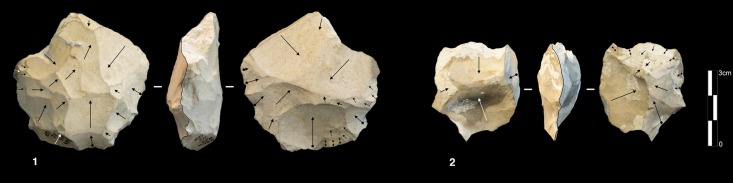
Bau de l’Aubesier. Discoid cores from sub-level K2.

This mode of production is only present in unit K, with five cores. Three of them are knapped on their total periphery (Discoid Type) and two cores show a partial exploitation (Fig 15).

**Fig 16:**
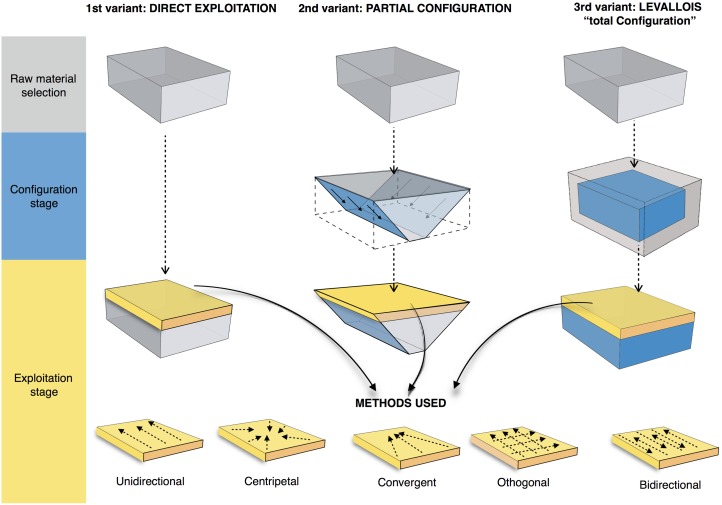
Bau de l’Aubesier. Variability of reduction systems in parallel planes exploitation.

**Fig 17:**
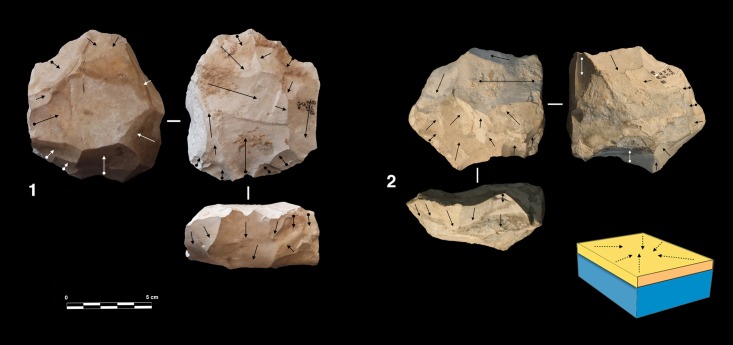
Bau de l’Aubesier. Levallois cores.

**Fig 18:**
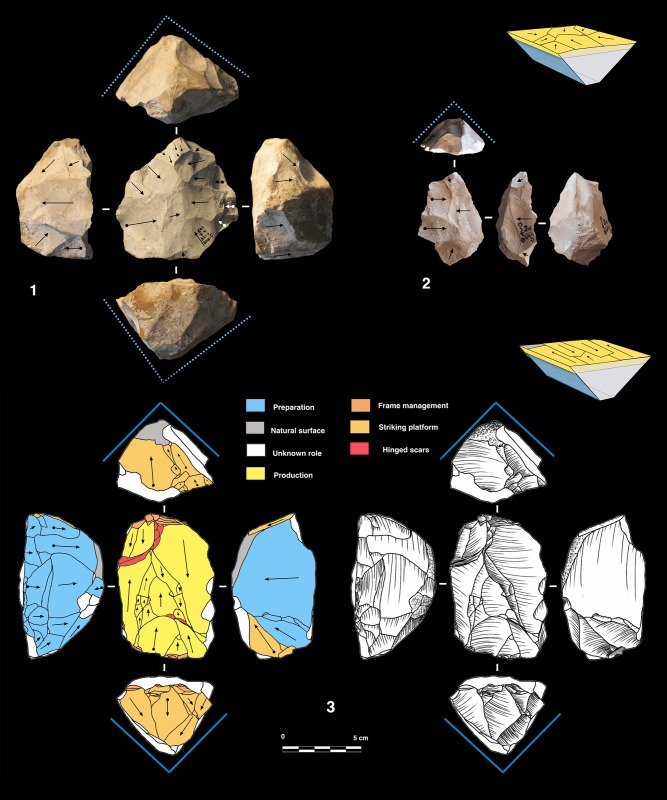
Bau de l’Aubesier. Cores with partial preparation.

Parallel planes exploitation is widely present. This category includes 24 cores from unit J and 6 cores from unit K. The methods employed are highly variable: centripetal, unidirectional, bidirectional, orthogonal and convergent. The bidirectional, convergent and orthogonal methods are found in both units K and J (Table 6). Conversely, the unidirectional method is only present in unit J, with 4 cores with a single series of removals and 6 cores with a multipolar exploitation. The centripetal method is primarily found in unit J, with just one centripetal core found in sub-unit K2 (Table 6). Three different types of configuration are recognized: Levallois, a partial configuration and a direct exploitation (Fig 16). Among the 30 cores, 6 of them, in unit J, can be described as Levallois (Fig 17). For the other 24 cores, two different processes in core management have been observed (Table 7). The first variant includes a preliminary phase that partially prepares the core by unidirectional removals that strike the two lateral surfaces. This operation gives the core a reversed trapezoidal cross-section (Fig 18). The aim is to create two lateral inclined striking platforms for the maintenance of the convexity on the flaking surface during exploitation. This particular process is mainly observed in unit K (Table 7). The methods are bidirectional, centripetal and orthogonal.

**Table 7:** Bau de l’Aubesier. Parallel planes exploitation; core variability.

System configuration	Methods	K2	K1	K	J4	J3	J2	J1	J	Tot.
**Direct exploitation**	Centripetal	0	-	-	1	-	1	-	1	3
	Unidirectional	-	-	-	1	1	1	1	-	4
	Bidirectional	-	-	-	1	-	-	-	-	1
	Multidirectional	-	-	-	5	-	-	1	-	6
	Convergent	1	-	-	1	-	1	-	-	3
	Orthogonal	-	-	-	-	1	-	-	-	1
	***Partial total***	***1***	***-***	***-***	***9***	***2***	***3***	***2***	***1***	***18***
**Partial configuration**	Centripetal	1	-	-	-	-	1	-	-	2
	Unidirectional	-	-	-	-	-	-	-	-	0
	Bidirectional	1	-	2	-	-	-	-	-	3
	Orthogonal	1	-	-	-	-	-	-	-	1
	***Partial total***	***3***	***-***	***2***	***0***	***0***	***1***	***0***	***0***	***6***
**Levallois**	Centripetal	-	-	-	1	2	-	-	3	6
	**Total**	**4**	**0**	**2**	**10**	**4**	**4**	**2**	**4**	**30**

**Fig 19:**
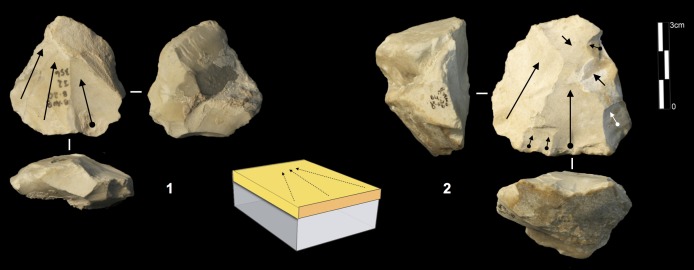
Bau de l’Aubesier. Convergent cores without preparation from Unit J.

Direct exploitation is based on the selection of a specific size and shape of raw materials, in order to avoid the first (configuration) phase. In this case, the exploitation of the core is preceded only by the preparation of the striking platforms. Exploitation is performed by unidirectional, bidirectional, centripetal, orthogonal and convergent methods. The convergent method is only used in the case of direct exploitation (Fig 19).

#### Volumetric exploitation systems

**Fig 20:**
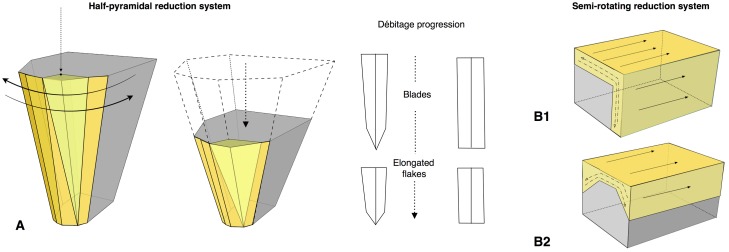
Bau de l’Aubesier. Variabilty in volumetric exploitation systems.

**Fig 21:**
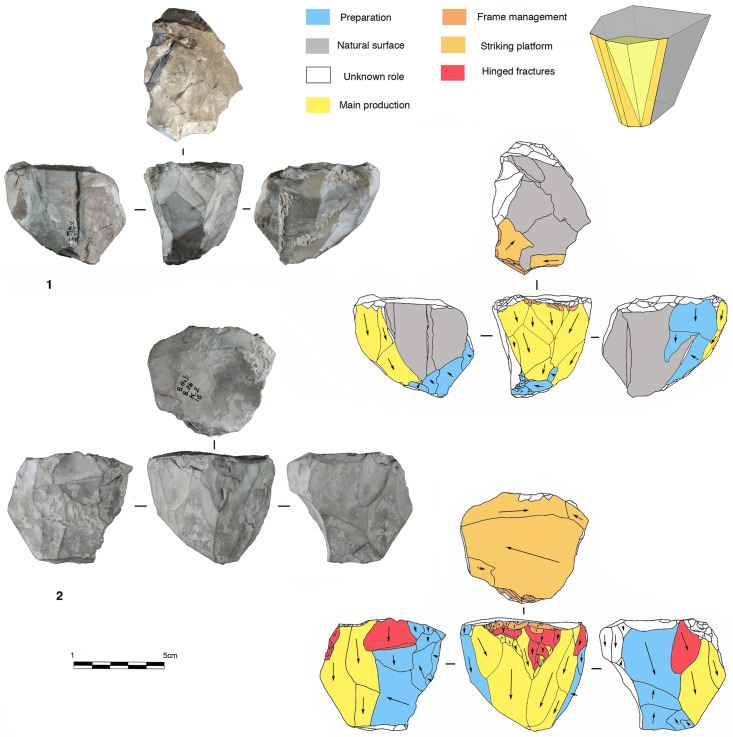
Bau de l’Aubesier. Half pyramidal cores.

Two types have been documented, for a total of 14 cores. Two cores are half-pyramidal cores and 12 cores are prismatic semi-rotating cores (Fig 20). The two half-pyramidal cores were found in unit K. Exploitation was carried out by convergent removals. In one case, the *débitage* starts from a cortical platform and shows a minimal phase of preparation in order to correct the distal convexity of the flaking surface (Fig 21 n.1). The second core shows a more elaborate re-configuration based on the re-centering of the flaking surface by lateral removals. After that, the core was abandoned after repeated hinged fractures (Fig 21 n.2). The semi-rotating system comes primarily from unit J, with just one core out of 12 from sub-unit K2. The core volume is not completely shaped out before starting blade production. The management of lateral convexities is performed by debordant blades. In rare cases a second opposite striking platform is used in order to manage the distal convexity.

**Fig 22:**
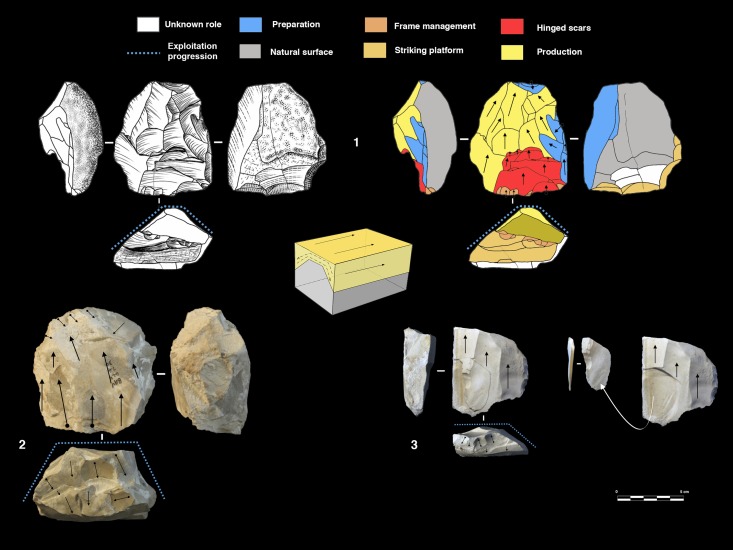
Bau de l’Aubesier. Semirotating cores. Sub-convergent core from sub-unit J4 (n.1); Unipolar core from sub-unit J4 (n.2); Refitting of unipolar semirotating core from sub-unit J2 (n.3).

**Fig 23:**
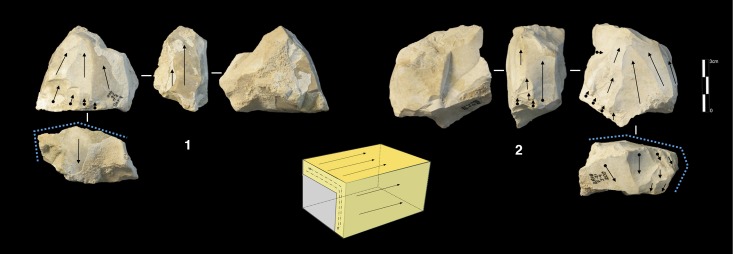
Bau de l’Aubesier. Convergent semirotating cores from sub-unit J4.

Removals can cover one (Fig 21 n. 3) or both of the lateral surfaces (Fig 21, n. 1, 2). The methods used are unidirectional and convergent (Figs 22 and 23).

#### Volumetric and parallel planes exploitation end products

**Fig 24:**
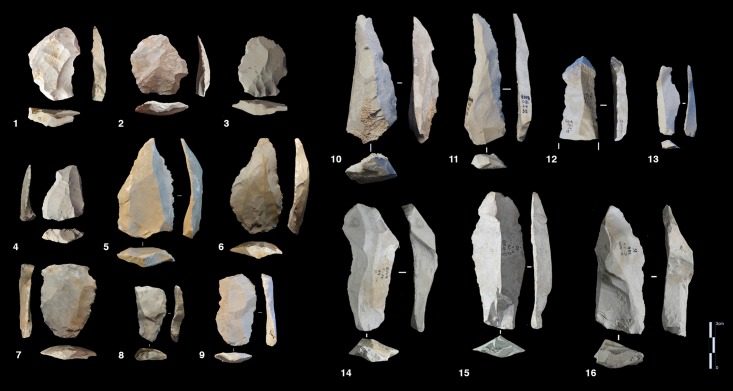
Bau de l’Aubesier. **End products.** On the left, flakes from parallel planes exploitation: centripetal flakes (n. 1 to 3), convergent flakes (n. 4 to 6), unidirectional and bidirectional flakes (n. 7 to). On the right, blades: convergent blades from unit J (n. 10 to 12) and from unit K (n. 16), unidirectional blades from unit K (n. 13, 14 and 15).

Core variability is similar to end-product variability. Centripetal flakes are the most frequent and are linked to the two main reduction processes (Table 8). Despite the low number of pieces, some observations can be suggested. Centripetal flakes with secant dorsal scars (type A) are present in units K and J but decrease over time (Table C in S2 File). Conversely, centripetal flakes with parallel dorsal scars (type B) increase in unit J, as do the equivalent cores. These products can be classified as Levallois-type flakes but can also be the results of three different processes: Levallois, direct exploitation, and partial preparation types (Fig 16). The variability of methods for parallel planes exploitation is confirmed by convergent, unidirectional and bidirectional Levallois-type flakes (Fig 24 n 1 to 9). Beside this dominant production of flakes, blades also exist in the two units; they are more numerous in the lower levels, with a proportion of 22.6% in sub-unit K2 and 7.6% in sub-unit J4. The blades are triangular or rectangular, consistent with the pyramidal cores, and the rectangular blades can be linked to the semi-rotating unidirectional system (Fig 24 n 10 to 16).

**Table 8:** Bau de l’Aubesier. Determined pieces. Numbers in brackets represent retouched pieces.

Levels	K2		K1-K		J4		J3		J2		J1-J	
	num	*%*	num	*%*	num	*%*	num	*%*	num	*%*	num	*%*
Flakes (Cortex >50%)	5	*6*.*0*	4	*3*.*7*	12(1)	*6*.*5*	3	*10*.*0*	2	*25*.*0*	10	*12*.*0*
Flakes (Cortex<50%)	11	*13*.*1*	-	*0*	20(1)	*10*.*8*	4	*13*.*3*	-	*0*	40	*48*.*2*
Centripetal flakes	13(2)	*15*.*5*	16(1)	*15*.*0*	39(11)	*21*.*1*	13	*43*.*3*	1	*12*.*5*	7(2)	*8*.*4*
Debordant flakes (chordal)	5(2)	*6*.*0*	5(1)	*4*.*7*	12	*6*.*5*	-	*0*	-	*0*	5	*6*.*0*
Unipolar flakes	10(1)	*11*.*9*	22(4)	*20*.*6*	30(4)	*16*.*2*	6	*20*.*0*	2	*25*.*0*	13(2)	*15*.*7*
Debordant unipolar flakes	3(1)	*3*.*6*	5(2)	*4*.*7*	4	*2*.*2*	-	*0*	-	*0*	1(1)	*1*.*2*
Bipolar flakes	4(2)	*4*.*8*	6(4)	*5*.*6*	2(1)	*1*.*1*	-	*0*	-	*0*	-	*0*
Debordant bipolar flakes	1(1)	*1*.*2*	-	*0*	1	*0*.*5*	-	*0*	-	*0*	-	*0*
Orthogonal flakes	-	*0*	-	*0*	1	*0*.*5*	-	*0*	-	*0*	-	*0*
Debordant Orthogonal flakes	-	*0*	-	*0*	1	*0*.*5*	-	*0*	1	*12*.*5*	-	*0*
Convergent/sub-convergent flakes	4(2)	*4*.*8*	5(1)	*4*.*7*	28(6)	*15*.*1*	2	*6*.*7*	-	*0*	1	*1*.*2*
Bladelet	1	*1*.*2*	-	*0*	-	*0*	-	*0*	-	*0*	-	*0*
Blades	19(4)	*22*.*6*	19(3)	*17*.*8*	14(5)	*7*.*6*	1	*3*.*3*	1	*12*.*5*	3(1)	*3*.*6*
Crested blades	2	*2*.*4*	1	*0*.*9*	-	*0*	-	*0*	-	*0*	1	*1*.*2*
Kombewa	-	*0*	3	*2*.*8*	1	*0*.*5*	-	*0*	-	*0*	-	*0*
Macro-tools	2 (2)	*2*.*4*	10(10)	*9*.*3*	1(1)	*0*.*5*	-	*0*	-	*0*	2(2)	*2*.*4*
Striking platform flakes	3	*3*.*6*	5	*4*.*7*	6	*3*.*2*	1	*3*.*3*	-	*0*	-	*0*
Shaping/retouching flakes	-	*0*	1	*0*.*9*	3	*1*.*6*	-	*0*	-	*0*	-	*0*
Rejuvenation flakes	-	*0*	2(1)	*1*.*9*	2(1)	*1*.*1*	-	*0*	-	*0*	-	*0*
Burin de Siret	1(1)	*1*.*2*	3 (2)	*2*.*8*	8	*4*.*3*	-	*0*	1	*12*.*5*	-	*0*
**Total**	84 (18)	*100*	107 (29)	*100*	185 (31)	*100*	30	*100*	8	*100*	83(8)	*100*

### Heavy-duty tools and retouched pieces

#### Payre

**Fig 25:**
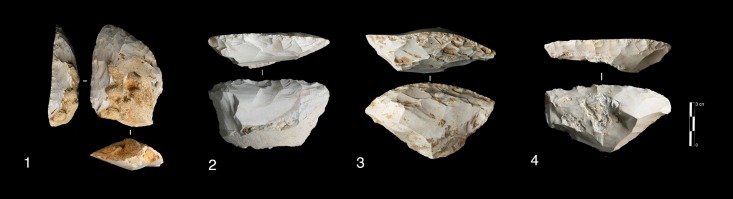
Payre. Quina tools from Unit G.

The lithic collections yielded denticulates, notches and sidescrapers (Tables 9, 10 and 11). The assemblages also include tools derived from shaping processes (the heavy-duty component) and some Quina pieces (Fig 25). The frequencies of tools in each assemblage range from 11.5% to 29.5% (Table 9). There does not seem to have been any specific choice of blank types from the *débitage* for any category of tool type (Table E and Table F in S2 File), except in unit G, where we observe more flake-tools from peripheral exploitation system blanks (centripetal flakes and debordant flakes).

**Table 9:** Payre. Proportions of retouched and unretouched pieces.

Levels	Gb		Ga		Fd		Fc		Fb		Fa	
	n	*%*	n	*%*	n	*%*	n	*%*	n	*%*	n	*%*
**Tools (flakes/handaxes/macro tools/pebbles)**	26	22.8	238	29.5	10	9.4	12	18.2	3	11.5	73	23.2
**Unretouched pieces (flakes/pebbles)**	88	77.2	568	70.5	96	90.6	54	81.8	23	88.5	241	76.1
**Total**	114	100	806	100	106	100	66	100	26	100	314	99.9

**Table 10:** Payre. Proportions of types of retouched pieces.

Levels	Gb		Ga		Fd		Fc		Fb		Fa	
	n	*%*	n	*%*	n	*%*	n	*%*	n	*%*	n	*%*
**Tools on flakes**	18	69.2	166	69.7	6	60.0	9	75.0	2	66.7	45	61.6
**Quina**	3	11.5	10	4.2	-	0	-	0	-	0	2	2.7
**Demi Quina**	2	7.7	17	7.1	1	10.0	-	0		0	6	8.2
**Handaxe**	2	7.7	-	0	-	0	-	0		0	1	1.4
**Partially shaped tools**	1	3.8	4	1.7	1	10.0	-	0		0	5	6.8
**Retouched pebbles**	-	0.0	41	17.2	2	20.0	3	25.0	1	33.3	14	19.2
**Total**	26	100	238	100	10	100	12	100	3	100	73	100

**Table 11:** Bau de l’Aubesier. Proportions of retouched pieces and blanks, excluding undetermined removals.

Levels	K2		K1-K		J4		J3		J2		J1-J	
	num	*%*	num	*%*	num	*%*	num	*%*	num	*%*	num	*%*
Retouched flakes	14	*16*.*7*	19	*17*.*8*	30	*16*.*2*	-	*0*	-	*0*	6	*7*.*2*
Shaped pieces	2	*2*.*4*	10	*9*.*3*	1	*0*.*5*	-	*0*	-	*0*	2	*2*.*4*
Unretouched flakes	68	*81*.*0*	78	*72*.*9*	154	*83*.*2*	30	*100*	8	*100*	75	*90*.*4*
**Total**	84	*100*	107	*100*	185	*100*	30	*100*	8	*100*	83	*100*

Most of the Quina tools were found in sub-level Ga (27 pieces). Predetermined reduction systems devoted to the production of large blanks for Quina retouch have been identified in the Middle Palaeolithic elsewhere [102, 103]. According to Baena [57], at Payre it is impossible to describe a Quina reduction process. The large and thick flakes used for Quina retouch can come from the first phase of secant parallel planes exploitation cores or from trifacial exploitation cores.

**Fig 26:**
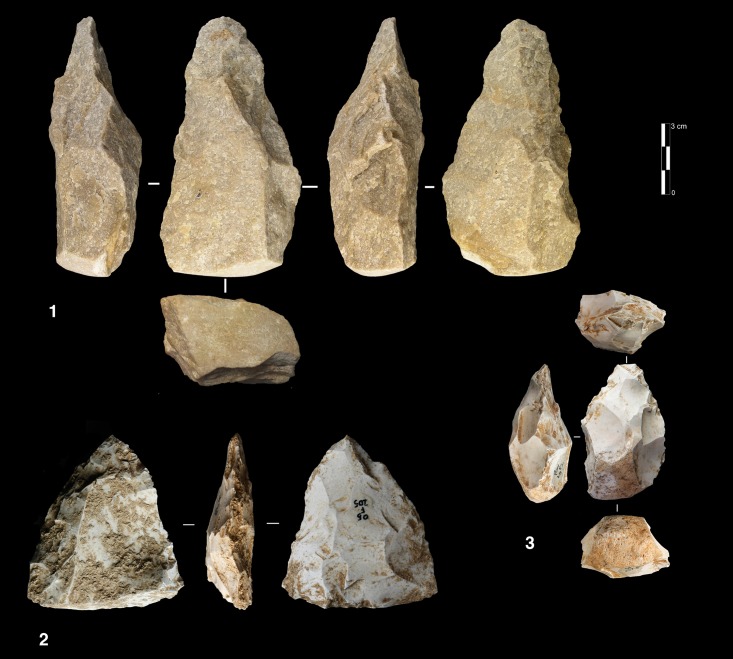
Payre. Bifaces from sub-unit Fa (n.1 and 2) and from Gb (n. 3).

Heavy-duty tools are rare. There are 3 handaxes, 2 in sub-level Gb and 1 in sub-level Fa (Fig 26).

**Fig 27:**
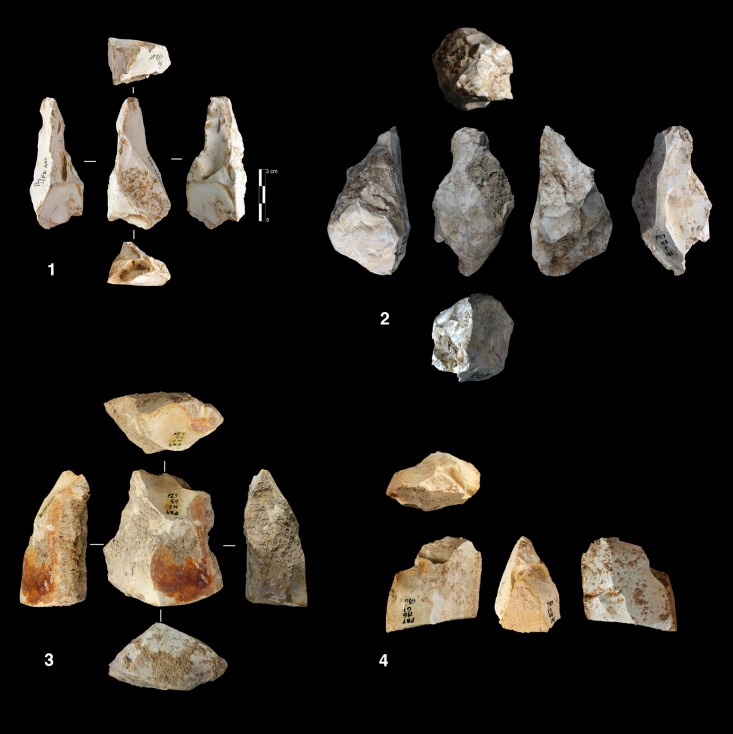
Payre. Partially shaped pieces from sub-units Ga (n. 1 and 2) and from Fa (n. 3 and 4)

Eleven tools are characterized by a partial shaping operation, aimed at creating a trihedral morphology while leaving the main part of the piece unmodified (Fig 27). Chi2 of 24,399 (df4) with alpha of 0,001 (18,467), indicates a significant difference between the levels for the proportions of retouched and unretouched pieces. This is also the case for the proportions of retouched pieces (Chi2 of 31,782, df = 12, with alpha 0,001, 32,909).

#### Bau de l’Aubesier

**Fig 28:**
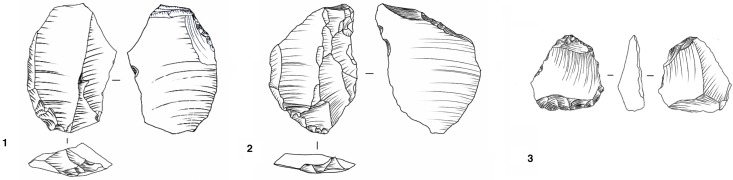
Bau de l’Aubesier. Truncated pieces from unit K (n. 1 and 2) and unit J4 (n.3).

**Fig 29:**
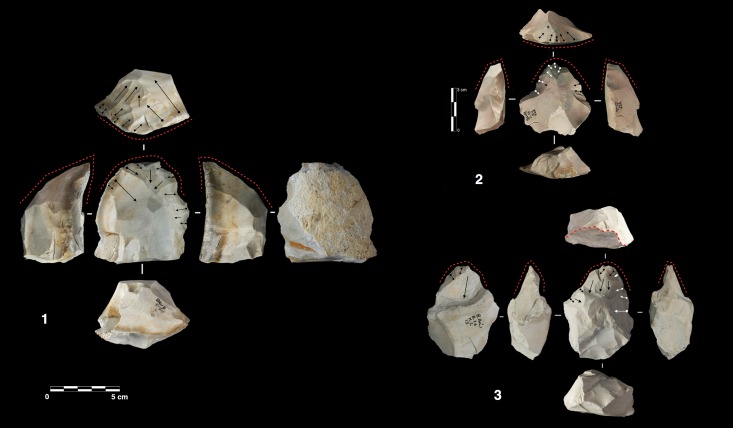
Bau de l’Aubesier. Partially shaped pieces from sub-unit K1 (n. 1 and 2) and sub-unit K2 (n. 3).

Retouched pieces are more frequent in units K (17.8%) and J4 (16.2%), but rare or totally absent in sub-levels J3 to J1-J. The retouch rarely modifies the form of the blanks, whether flakes or blades. The only exception concerns 14 truncated pieces in unit K (12 pieces) and in sub-unit J4 (2 pieces) (Fig 28). Fifteen pieces (12 from unit K and 3 from J) are characterized by partial shaping to build a rostrum (Fig 29). The rest of the piece is unmodified, except in the case of one piece from sub-level K2 (Fig 29 n. 3). Within the production no specific blank type was selected to be retouched (Table D in S2 File).

The Chi2 of 30,714, with df = 8, indicates a significant difference between the levels in term of ratios of retouched pieces (alpha 0,001, 27,877).

## Discussion and Conclusion

### Similarities and differences in lithic production at Payre and the Bau de l’Aubesier

**Fig 30:**
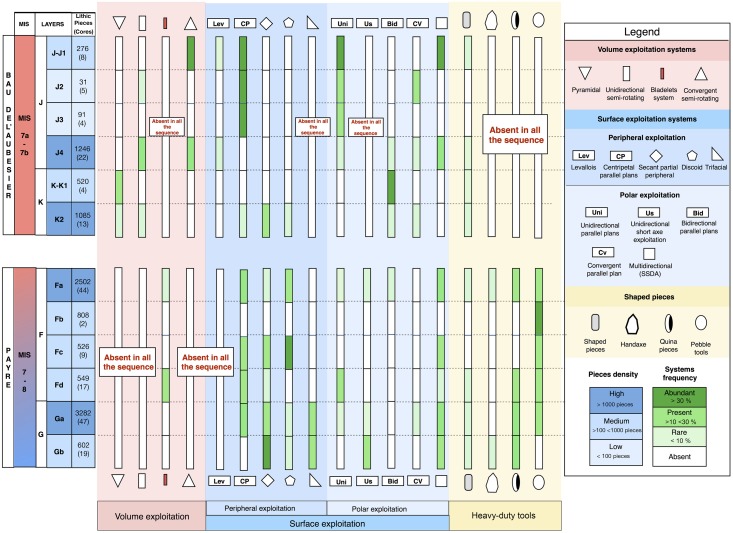
Summary of the reduction processes over the sequences at Payre and the Bau de l’Aubesier.

The technological strategies performed at the Bau de l’Aubesier and Payre show both differences and common features over time. At the Bau de l’Aubesier the major differences between units K and J include the appearance of Levallois *débitage* in unit J, in parallel with the disappearance of Discoid production. The pyramidal system disappears in unit J, replaced by the development of a semi-rotating system and in particular by the convergent method, which is absent in unit K (Fig 30). At Payre, the differences between the sub-levels seem to be less marked than at the Bau de l’Aubesier. The main shift over the time span from unit G to unit F is constituted by an increase of the Discoid system, associated with a decrease in the partial peripheral system and the disappearance of the trifacial cores. The shift also includes a marginal bladelets production and a decrease in the number of Quina pieces.

Comparing the lithic assemblages of the two sites, differences in terms of technological behaviours appear clearly at multiple levels: core management, reduction systems and tool kits. From a macroscopic point of view the Payre assemblages are the result of a double behaviour, with both knapping and shaping processes (Fig 30). Shaping processes are almost entirely absent at the Bau de l’Aubesier, represented only by core technologies and the partial shaping operation described for a few pieces. If we compare the core technologies between the two sites, strong and clear differences appear. At the Bau de l’Aubesier, reduction systems were performed on both the surface and the volume of cores, in order to produce both flakes and blades. Conversely at Payre, volumetric exploitation is absent except for a marginal but noteworthy bladelets production (Fig 30).

If we compare the exploitation systems in detail, differences are again recognizable. The Levallois concept present at the Bau de l’Aubesier is totally absent at Payre. Flakes at Payre are mainly produced by secant planes exploitation, discoid systems, partial peripheral systems and trifacial cores. At the Bau de l’Aubesier, variability in flake production is principally due to parallel planes exploitation systems, including Levallois, while exploitation by secant planes plays a minor role.

Concerning tool composition and proportions, we again observe different trends at the two sites. Primarily, the tool kit proportion is higher at Payre than at the Bau de l’Aubesier, ranging from 11% to 29.5% at Payre, while at the Bau the l’Aubesier, tools represent between 7.2% and 17.8%, and in sublevels J3 and J2 they are totally absent. At Payre, the flake-tools are notches, denticulates and scrapers. Moreover, some Quina pieces, pebble-tools and some handaxes make up the heavy-duty component. At the Bau de l’Aubesier, the tools are on flakes and blades and are retouched by a marginal retouch that only slightly modifies the pieces, except for some truncated pieces and some partial bifacial tools (rostrum). This reduced importance of the tool kit at the Bau de l’Aubesier can be explained by the use of core technologies based on predetermined systems, such as Levallois, which produce a blank form that does not need to be modified.

The common features between the sites are minor. The two sites share the use of unidirectional methods by parallel planes exploitation, even if at the Bau de l’Aubesier some types are absent. The main common features concern Payre units G and F and Bau de l’Aubesier unit K, where the Discoid system and the secant partial peripheral system were both found.

### Possible reasons for the variability between the lithic assemblages of the two sites

Current archaeological data show a great versatility in Neanderthal technological behaviour. As we discussed in the introduction, the archaeological literature has for decades reflected the difficulty of relating this diversity to clear general, regional or local cultural packages. Traditions, land use and mobility, types of activities, duration of occupation, and sometimes demography, have been proposed, alone or in various combinations, as possible explanations for this variability over space and time. Given that we have only a few sites of Early Middle Palaeolithic (EMP) age (from the end of MIS 8 to MIS 7) in southern France, we have no evidence of changes in local demography. However, we can, we can at least examine the raw material availability and modes of procurement (land use and mobility), and the subsistence strategies (including site use and activities) reflected in our site assemblages, in order to try to better understand the variability in human behaviour during this time period.

The two sites which are the subject of this present study are located in a similar environmental setting, within the same region in south-eastern France, being (broadly speaking) located on opposite sides of the Rhône corridor. They are both in a more or less open cave or rock shelter, opening onto a slope of a narrow valley with a river, and close to low plateaus. Payre is closer to the Rhône Valley, while the Bau de l’Aubesier’s environment is dominated by the nearby Mont Ventoux (1912 m elevation). Nonetheless, it is reasonable to assume that deposits that accumulated during the same time period at the two sites would have accumulated under similar conditions of climate and floral and faunal resource availabilities.

Despite this environmental similarity, however, the technological strategies and tool kits differ greatly, and few common features can be observed between the occupations of the two sites. We also see that at each site, there are differences between the layers, showing change in technological approaches through time. This diversity of strategies is therefore clearly not only due to the particular site but also reflects variability in strategies employed by the human groups living in this part of France at that time.

#### Raw material strategies

Flint procurement patterns at Payre indicate differences in land use through time, perhaps due to differences in the duration of occupation between units G and F. Unit G is considered to have recorded long-term occupations while unit F reflects short-term occupations in a smaller cave (based on meso- and micro-wear studies on ungulate teeth) [104, 105]. For instance, for sub-level Gb, 11 flint types have been described [106, 107]. Most of the flint came from the southern plateau along the Rhône River, following a North-South axis. Flint was collected mainly on primary formations or at secondary formations such as fluvial deposits, located from 5 to 15 km around the site, as fragments of nodules or large flakes. Some large flakes and nodules came from 30 km or small flakes from 60 km to the south of the site (reflecting partial reduction processes). The Rhône valley itself was rarely used for flint procurement. Conversely, in Unit F most of the flint was collected from alluvial deposits (90%). The exact location of the outcrops is therefore impossible to identify and only a maximum perimeter may be given. Ten types have been identified, some of which are also found in the unit G assemblage. Flint collecting was carried out more to the west of the site than is the case for unit G, but there was again some collecting in the southern area.

The basalt in the two basal units was collected at the foot of the site, from the remains of terraces on the slope. Primary sources of basalt are located upstream of the Payre River in the volcanic massif of the Coiron. Basalt was introduced as pebbles of various sizes or as large flakes; most of the pebbles were left whole. Despite the badly preserved superficial surfaces of the pebbles, some show percussion marks and could have been used as hammerstones. The elongated and flat pebbles were shaped into unifacial pebble-tools and left numerous cortical flakes. Crushing marks and flakes from rejuvenation attest to their use *in situ*.

Local quartz arrived as rare pebbles and above all as flakes. The reduction processes are partial [107]. The rare cores have two secant or orthogonal surfaces. As was the case for the flint, flaking was mainly performed by a series of unipolar removals, rarely centripetal. Some large pieces could be modified cores (crush marks on the cutting edges) or are large tools on fragments of pebbles. The flakes are thick and sometimes backed. Between 10 and 15% are retouched (one edge or convergent edges).

The marly and siliceous limestones were collected in the Payre or Rhône Rivers. Some fragments of the cave limestone were collected and just retouched. Pebbles were broken or shaped. Flakes are numerous, thin, largely cortical and small, and imported, as for quartz. Few are retouched. Two limestone cores in level Fa cannot be refitted with flakes. The flaking took place on small flat pebbles and cores with two secant surfaces.

Quartzite arrived as pebbles and above all as large and small flakes, collected possibly along the Rhône River. The large flakes, flaked from large cobbles outside of the site, are unretouched or retouched as large unifacial or bifacial tools (peripheral, pointed or transversal). The large flakes are cortical or partially cortical. Crushing marks on edges support a use for heavy activities. Only one piece could be considered as a core or a re-used broken bifacial tool. Small flakes could come from the rejuvenation of the heavy-duty tools or have been imported for unknown reasons, as with the large basalt tools on flakes.

At the Bau de l’Aubesier, almost the entire assemblage is in flint, but it has been possible to distinguish many different types of flint and track them back to source areas throughout the region, as well as evaluate a variety of characteristics of each source area that would influence hominins’ choice of whether or not (or how much) to use it [108, 109, 110, 111, 112, 113, 114]. In all levels, a considerable proportion of the lithic assemblage has been patinated to such an extent that the pieces can only be identified as being flint, and no source can be attributed. There are also a few flint types for which sources have not been identified, but these account for a very small number of pieces. Taking these together with the patinated pieces, however, in levels J-J4 together, the sources of 43.2% of the pieces are either unidentified or unidentifiable; in levels K-K2 these account for 51.7% of pieces.

**Fig 31:**
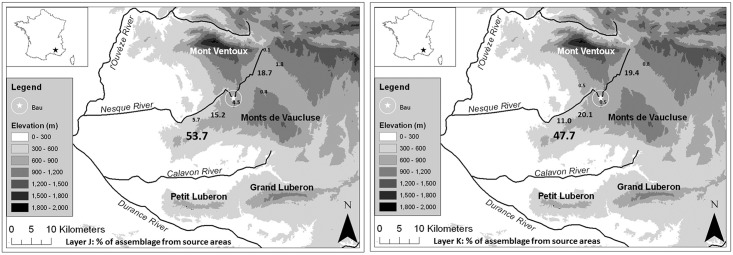
Bau de l’Aubesier. Map of sources of flint in layer J (on the the left) and K (on the right).

Once these unidentified pieces are excluded, the small numbers of remaining pieces in the sub-layers make it more reasonable to combine sub-layers and deal with an overall layer J and an overall layer K. These two assemblages have similar sizes: 830 pieces in J, and 880 in K. A variety of attempts has been made to try to detect whether the patination of such a large number of pieces has biased the remaining sample (e.g., with patination affecting some flint types more than others, thereby selectively removing them from the sample). No such effect has been found, so we consider these samples to be representative of the overall use of raw material sources for each assemblage. In these layers, all of the material was obtained within 15 km of the site, with the bulk of it coming from source areas along a SW-NE trending axis (Fig 31).

In both layers, the assemblages are dominated by material from the Murs area, to the SW of the site, despite the fact that this is at the far end of the normal provisioning range (Table 12). Clearly, a distance-decay model, where raw materials would be less common the farther away their source is, does not apply in this case. There are some differences between the two layers in terms of the percentages of material from Murs among various typological categories, but in both layers all parts of the *chaîne opératoire* are present and common, suggesting that this raw material was imported as nodules or cores, and worked *in situ*.

**Table 12:** Bau de l’Aubesier. Sources of raw material in the assemblages.

		Layer J		Layer K	
Source Area	Direction	n.	*%*	n.	*%*
Nord Aurel	NE	1	*0*.*1*	-	*0*
St. Trinit	NE	15	*1*.*8*	7	*0*.*8*
Sault	NE	155	*18*.*7*	171	*19*.*4*
Nord des gorges	NW	-	*0*	4	*0*.*5*
St. Jean de Sault	E	3	*0*.*4*	-	*0*
Local	--	37	*4*.*5*	4	*0*.*5*
Faraud	SW	126	*15*.*2*	177	*20*.*1*
Méthamis	SW	47	*5*.*7*	97	*11*.*0*
Murs	SW	446	*53*.*7*	420	*47*.*7*
**TOTAL**	--	830	*100*	880	*100*

Raw material from other sources to the SW is also common in both layers, such that overall 74.6% of pieces in layer J, and 78.8% of pieces in layer K, are from the SW area. Use of sources off the SW-NE axis is extremely minor, and variable. Use of the source closest to the Bau de l’Aubesier is also minor, especially in layer K (only 4 identified pieces), and we have as yet no explanation to suggest for that.

The use of sources to the NE (which include the one piece of quartzite) does follow a distance-decay pattern, and the overall percentage of pieces from those sources is very similar in the two layers (20.6% in layer J, 20.2% in layer K). There are however some noteworthy differences in the use of these materials for different typological categories. In the lowest layers, K-K2 together, the material from the NE is distributed among tool categories in much the same way as the material from the SW, suggesting that it, too, was imported and knapped *in situ*. In layer J, however, the material from the NE is more common among the retouched flakes, large flakes and blades than it is in the debris, small flakes or cores, suggesting that it was imported in a more finished form, representing a more specialised or careful use of that material than the material from the SW. For example, taking retouched and shaped items together, raw material from the NE makes up 27.3% of such items in layer J, but only 13.7% of them in layer K. On the other hand, material from the NE accounts for only 4.9% of cores in layer J, but 13.6% of cores in layer K. We can therefore propose that strategies for use of the landscape, reflected in the lithic assemblages, varied through time at the Bau de l’Aubesier.

#### Subsistence strategy

At Payre the spectrum of ungulates is mainly composed of red deer (*Cervus elaphus*), horse (*Equus mosbachensis*), bovines (*Bos primigenius* and *Bison priscus*) and rhinoceroses (*Dicerorhinus hemitoechus* and *D*. *kirchbergensis*). Carnivores are especially numerous in unit F. Among them, cave bear (*Ursus spelaeus*) is predominant, associated with other carnivores including some large predators such as wolf (*Canis lupus*), hyena (*Crocuta spelaea*) and cave lion (*Panthera (Leo) spelaea*) [115, 116, 117, 105]. This faunal list reveals a mildly cold climate and different biotopes, including forests, wooded prairie, steep rocky sides (Payre canyon), as well as open-steppe environments. The microfaunal remains indicate colder and steppic environments in units G and F [118, 119].

The occupation types were different in units G and F. In F, carnivores commonly inhabited the site, suggesting that hominid occupations alternated with carnivore denning [116, 105]. The study of the ungulate tooth microwear patterns attests to longer occupations in a larger cave in unit G than in unit F, where the cave’s size and ceiling height were reduced, in agreement with the smaller number of lithic artefacts and the taphonomical study of the faunal remains. Unit F was mostly a carnivore den with shorter-term human occupations [104, 105]. Unit G recorded longer-term occupations with a high anthropogenic impact on horses, deer and bovids, the three main hunted species [116].

The anthropogenic activities left numerous types of evidence at Payre. Ungulate bones were intensively cut-marked, broken, and some were burned. Fire was used in each layer, but there are no clear hearth structures other than in unit G. The lithic residues and use-wear analysis show evidence, among other things, of fish processing in units Fa and D and of the use of avian resources [120].

Similarities in the faunal corpus exist between units G and F [121, 116, 67, 105]. The main species which characterize the assemblages are cervids, bovines, horses and rhinoceros. The rhinoceros remains include only young and old individuals, but the three main species are represented by adults, young, and young adults. In unit G, mortality curves indicate hunting all through the year. In unit F, conversely, hunting was more frequent in the autumn. In the two units, rhinoceros remains are considered to be possibly mainly due to scavenging (since they consist of a few fragmented long bones and teeth), although for unit F there is some evidence of occasional hunting: 5 adult and young individuals are represented by more bones from the whole carcasses. These were probably obtained in the swamps of the Rhône Valley at the foot of the cave.

The difference between the two units is mainly due to the action of carnivores. In both units, faunal remains indicate the main anthropogenic accumulations. In unit F carnivores played a large role in the consummation of carcasses, and bear occupations were important: tooth marks indicate that these were secondary occupations after the departure of the humans. In unit F, carnivore tooth-marks are present on between 2 and 6% of the NR > 5 cm, but in unit G, the value is around 1%. Except for cave lion and wild cat, the same species of carnivores exist in the two units. Unit F is moreover largely dominated by remains of *Ursus spelaeus*. Bears settled in the cave for winter, alternating with human occupations; these are followed in abundance by wolves, hyenas and big cats using the site. In unit G, cave bears are less numerous, as are foxes, hyenas and wolves.

Lack of cut-marks on the different taxa of herbivores indicates that some small species were not brought by humans to the cave (roe deer, tahrs or boars). The middle- and large-sized herbivores attest conversely to human actions, which were more intense in unit F (cut-marks and bone breakage for marrow recovery). In both units, the anatomical proportions of ungulates and location of anthropogenic marks indicate primary butchery activities for cervids and secondary butchery activities for bovines, horses and rhinoceros (with the first skinning having taken place at the hunting location).

Burnt bones in the two units provide evidence of fire use, with the possible use of bones as the combustible. In unit G, one ash lens could be the remains of a fire place. Some bone retouchers attest to the use of bone [68].

At the Bau de l’Aubesier, Fernandez reports for layers J and K combined that the bones are highly fragmented, but there are abundant teeth. He was able to identify a minimum of 38 individual animals. Most of these were of large animals: 17 *Bos primigenius*, 12 *Equus mosbachensis*, 1 *Dicerorhinus hemitoechus*, and 1 *Megaceros giganteus*. There was also 1 *Cervus elaphus*, 2 *Capreolus capreolus*, 2 *Hemitragus cedrensis*, 1 *Dama dama*, and 1 *Rupicapra rupicapra*. From this, he suggests that these lowest levels are probably from MIS 7 or early 6, and that the climate was rigorous, with an open landscape. The two main species hunted, aurochs and horse, were both large animals but with very different behaviours, necessitating two separate hunting strategies [122, 123, 124, 125]. The seasons of death of the ungulates indicate use of the site all throughout the year, suggesting either that the assemblages consist of palimpsests of several short-term occupations, or that they are due to long-term occupations. There is little or no sign of carnivore activity, or of the use of fire by the hominins. Given the poor state of the bones, it is unclear how much butchery was done at the site.

### Payre and the Bau de l’Aubesier in the MIS 9–7 European context: Traditions?

The comparison between Payre and the Bau de l’Aubesier shows differences in technology, but does not show any significant divergence in terms of raw material strategies. At both sites, we see a local and semi-local provisioning with good quality flint, and some other local rocks. Raw materials were collected in the form of nodules, pebbles, flakes and slabs. Flint was largely employed for flaking at Payre even if a minor quantity of quartz was used as well. At the Bau de l’Aubesier the raw material used was flint except for one piece of quartzite. For both levels considered at the Bau, flint was procured within 15 km of the site, preferentially along a NE-SW axis. At neither site do we see any undeniable relationship between changes through time in core technologies or tool kits and mode of flint procurement. At Payre, however, local stones (basalt and quartzite) were shaped to produce bifaces and pebble tools. These types of tools are absent at the Bau de l’Aubesier.

How can we account for these technological differences? There are no signs of different specialized activities at either site. The sites are, for instance, not solely butchery sites. The subsistence strategies at both sites show that faunal resources were treated and consumed *in situ*. Herbivores were the main species hunted and each site is characterized by both short and long-term seasonal occupations. Site function can be indirectly related to intensity of tool use, but in both cases here we find limited retouch, rarely modifiyng the shape of the blank. This type of rejuvenation does not indicate an intense site use, but instead reflects choices in tool kits. Micro-wear studies at Payre confirm this, indicating that multiple domestic activities were performed on the site whatever the duration of occupations [57, 120]. Thus the raw material procurement and subsistence strategies do not account for the technological differences observed between Payre and the Bau de l’Aubesier, nor for changes between levels at either site.

This then seems to bring us back to the debate of Bordes vs. Binford on the importance of the role of activities vs. cultural traditions. Unfortunately, with so few known sites of this age, any changes in demography that can have affected the area are unknown, and whatever they may have been, they are unlikely to have influenced the types of reduction strategies practiced in each level of a single site. Still, whether or not populations increased during this period of time, it is probable that relationships between Neanderthal groups on the two sides of the Rhône River were limited. The river would have been hardly passable most of the time, and would have been a formidable natural barrier between regions.

To evaluate the idea of traditions, the technical behaviour observed at Payre and the Bau de l’Aubesier must of course be seen in the context of the variability of the Western European EMP (Table G in S3 File). The coexistence of some handaxes and dominant standardized core technologies in the Payre sequence is a typical pattern of the EMP, with persistence of residual bifacial tools in some sites as late as MIS 6 [12, 126, 127, 128, 129, 130, 131]. The presence of the Levallois core technology at the Bau de l’Aubesier is another technological feature shared by similarly-aged assemblages. On the other hand, some particular technological features characterise each assemblage. The volumetric blade production at the Bau de l’Aubesier, dated to the end of MIS 7, provides evidence that this type of technology is older than previously shown in southern France, where until now it had been dated to the end of MIS 6 and the beginning of MIS 5 [42, 45, 46, 47]. In a broader comparison, the only other site in southern Europe with a blade production earlier than MIS 5 is Cave d‘Olio located in northern Italy, which dates back to MIS 9 [10, 132]. As at the Bau de l’Aubesier, at that site there is also early evidence of Levallois core technology. Moreover, the semi-pyramidal cores related to the blade production at Cave dall ‘Olio and partially at the Bau de l’Aubesier are unusual for the EMP, where the most common reduction systems are linked to prismatic cores exploited through a rotating and semi-rotating rhythm.

The production of bladelets at Payre, even if uncommon, is another noteworthy behaviour which is rare in the EMP. The intentional production of bladelets is not commonly recorded until the final phases of the Middle Palaeolithic (MIS 4–3). It has been noted at the sites of El Castillo and Cueva Morin in Spain [133], at Champ Grand [134, 135] and Combe Grenal in France [136], at Fumane and at Grotta del Cavallo in Italy [137, 138, 139], and at Balver Höhle in Germany [140]. Recently, a bladelet production dated back to MIS 5 has been described at the site of Riparo del Molare in southern Italy [141].

Focusing our comparison on south-eastern France, where Payre and the Bau de l’Aubesier are located, a more few sites can help us to propose a regional scenario. The presence of the Levallois technology in south- eastern France during the EMP is well known. The sequences of Orgnac 3, covering a span time from MIS 9 to 8, show a gradual development of Levallois technology with a decrease in bifacial tools [142, 143, 144]. The association of a few bifaces and bifacial tools and dominant core technologies brings together Orgnac 3 and Payre, except that the Levallois technology is lacking at Payre (except for some possible pieces introduced into the site in unit F).

The blade production observed at the Bau de l’Aubesier may be compared to what is described at Baume Bonne with both Levallois and blade technologies dated to the MIS 6/5. The sequence of Baume Bonne, dated from MIS 10 to MIS 5, is long and complex, with changes in technical behavior through time [145, 146]. The early phases, units I and II (MIS 10 to 8), show a coexistence of bifaces and pebble tools with a production of flakes by discoid and SSDA technologies. In MIS 8, the Levallois technology is present and is associated with rare bifaces. During the MIS 6 and 5, the Levallois is stabilized and diversified in various methods including the production of blades [46]. The lack of Levallois evidence associated with shaping processes in the earliest phase at Baume Bonne constitutes a trend comparable to what is recorded in units G and F at Payre, while the development of the Levallois and blade technologies in the recent phases at Baume Bonne only partially corresponds to what is recognized at the Bau de l’Aubesier. The Levallois core technology at Baume Bonne is performed by various methods (convergent, centripetal, unipolar) while at the Bau de l’Aubesier only the centripetal method is employed. Moreover, at the Bau de l’Aubesier, blade production is exclusively made by volumetric systems (pyramidal and prismatic) while at Baume Bonne, blades are obtained by both a volumetric and a surface management (Levallois) system. This is also the case for Baume Flandin, close to Payre and dated to the MIS 5e [42, 44], with blade *débitage* by a Levallois concept and a debitage directly on flint slabs. This *débitage* is associated with a Levallois flake technology in the same level. Thus we see a complex situation, where some of the technical characteristics of Payre resemble those found at other sites around the region, although not always in deposits of the same age, and some of the technical characteristics of the Bau also resemble those found at some of the same sites, but at different times, in addition to possible resemblances between the Bau and still other sites. There does not seem to be a simple pattern emerging, which we could attribute to any one or several factors.

## Conclusion

Technological behaviours recognized at Payre and the Bau de l’Aubesier shared features typical of the broader EMP such as, on the one hand, the presence of handaxes, and on the other the use of Levallois and laminar core technologies. However, differences between the sites appear in the reduction systems employed (volumetric and Levallois concepts only observed at the Bau de l’Aubesier), types of end-products and tool kits. This variability does not seem to be linked to external factors such as the raw materials or other activities. The two sites are located within the same region, on opposite sides of the Rhône River valley, so their environments would have been similar, and we could expect more common features between them. It may bethat this particular geographical situation—on opposite sides of a major river—is in fact one of the reasons which contributed to maintaining distinct technological traditions even if the sites are contemporary. The results at Payre and the Bau de l’Aubesier are an excellent illustration of the diversity of technological strategies employed by the human groups of the EMP. They demonstrate that the trajectory of behavioural changes in material culture is far from homogeneous and monolithic in time and space. Depending on the chronological and geographical scale, the classical subdivision between Lower and Middle Palaeolithic must be revised to describe a complex and multifaceted archaeological reality with a rhythm which remains to be described. The debate about the meaning of the diversity of Middle Palaeolithic assemblages, Early or Late, is far from over.

Finally, we suggest that the EMP should be understood in terms of its diversity of strategies, the use of which fluctuated through time and across space, rather than having certain sets of behaviour tied to certain regions, or, by extension, to the “cultural groups” that might have lived there.

[147]. The EMP phase may predate the development of regional cultural similarity and technological behaviours in the Late Middle Palaeolithic, which favoured intragenetic geneflow and the emergence of the classical Neanderthals [148].

## Supporting information

S1 File Materials and methods. (DOCX)

S2 File Table A. Payre, type A and type B flakes. Table B. Payre, type of platform of type A and B flakes. Table C. Bau de l’Aubesier, type A and type B flakes. Table D. Bau de l’Aubesier, type of platform of type A and type B flakes. Table E. Payre, comparison of the flake techno-types with the incidence of retouch for each category. Numbers in brackets indicate the number of retouched pieces for each category. The % ret column indicates the percentage of retouched pieces for each category. Table F. Bau de l’Aubesier, comparison of the flake techno-types with the incidence of retouch for each category. Numbers in brackets indicate the number of retouched pieces for each category. The % ret column indicates the percentage of retouched pieces for each category.
(DOCX)

S3 File Table G. Data of European sites from MIS 9 to 7. Payre and Bau de l’Aubesier are in bold. Certain technological identification (X). Uncertain technological identification (X?).
(DOCX)
