# Supplementary material for: Technological variability during the Early Middle Palaeolithic in Western Europe. Reduction systems and predetermined products at the Bau de l'Aubesier and Payre (South-East France)
Source: PLoS One. 2017 Jun 7;12(6):e0178550. doi: 10.1371/journal.pone.0178550 (PMC5462386; doi:10.1371/journal.pone.0178550)
Supplement: S1 File — (DOCX) [file pone.0178550.s001.docx]

**Technological variability during the MIS 9-7 in Western Europe. Reduction systems and predetermined products at the Bau de l’Aubesier and Payre (South-East France).**

**Supporting Information**

**Supplementary File S1**

**Materials and Methods**

This PDF file includes:
Lithic analysis terminology

References

To clarify the terminology used in this work we are going to describe it in the technological order used during the study of the lithic collection.

Volumetric concept: Volumetric and Surface exploitation were distinguished by means of the volumetric structure analyses (Boëda 1988, 1990, 2013). Supplementary differentiation was integrated to distinguish a surface exploitation applied on a large surface as in the case of the Levallois concept and a surface exploitation applied to a narrow surface but that exploits the core along its short axis. We limited the attribution to a volume exploitation just to the cores that are exploited on the narrow surface along its longer axis.

Striking platforms organization: In relation to the organization of the striking platforms and their positioning on the core the reduction process can follow two types of exploitation modality: polar and peripheral. The polar modality includes all of the reduction systems based on the extraction of a series of removals that are detached starting from a striking platform positioned at one of the poles of the core. In relation to the position of the striking platforms on the cores, three categories of polar exploitation can be identified: Unipolar, Bipolar with opposite striking platforms, and Bipolar with orthogonal striking platforms. In this subdivision, we do not consider multipolar exploitation to be a valid variant, unlike SSDA systems. In fact, in that case the presence of more than two series of removals on different surfaces of the core can be the result of the repetition of the unipolar or bipolar modality on the same volume.

The peripheral modality includes the reduction systems where the removals were struck from platforms extending around the core’s periphery without a specific starting pole. This category includes Discoid systems (unifacial and bifacial) and the partial peripheral exploitation systems.

Exploitation methods: After the choice of the volumetric concept and the positioning of the striking platforms (prepared or not), the exploitation method chosen is another technological variant, which serves to complete the reduction system. We defined the method as an algorithm that is a minimal sequence of detachment organized in relation to the direction of the removals and their combination. The direction of the methods can be convergent, unidirectional, bidirectional, centripetal, chordal, or orthogonal. On the basis of the combination of the removals, the bidirectional method can follow an intersected or alternated rhythm. Various other combinations of removals are possible.

The organization of the detachments was identified by taking into account a previous diacritical analysis of both cores and flakes, distinguishing the initial stage from the main stage of the *débitage*. Based on this we attributed each piece to a specific method, taking into consideration just the scars that are correlated with the main stage of *débitage*.

Angle of exploitation: Whatever the reduction systems used the direction of the exploitation of the core can have just two variants: by secant plans and by parallel plans. Among the main reduction systems, exploitation by secant plans is common in the discoidal systems and partially used in Quina reduction systems. The exploitation of parallel plans is used for the majority of the reduction systems, including the Levallois concept. The inclination of the scars of the negatives of the removals has also been documented in order to distinguish whether the blanks come from a secant or parallel plans exploitation. Measurement of the angle degree for both cores and blanks was made with a profilometre.

**References**

Boëda E. (1988) Analyse technologique du débitage du niveau II A. In: Le gisement paléolithique moyen de Biache-Saint-Vaast (Pas-de-Calais), vol. 1, Tuffreau, A., Sommé, J., Editors, Mémoire de la Societé Préhistorique Française 21, Paris, pp. 185-214.

Boëda E. (1990) De la surface au volume: analyse des conceptions des débitages Levallois et laminaire. In: Paléolithique moyen récent et Paléolithique supérieur ancien en Europe. Ruptures et transitions: examen critique des documents archéologiques, Farizy, C., Editor, Mémoires du Musée de Préhistoire d’Ile de France, Nemours, pp. 63-68.

Boëda E. (2013) Techno-logique & Technologie. Une paléo-histoire des objects lithiques tranchants. Coll. Préhistoire au Present, Archeo-editions, 259 p.
