# Supplementary material for: Technological variability during the Early Middle Palaeolithic in Western Europe. Reduction systems and predetermined products at the Bau de l'Aubesier and Payre (South-East France)
Source: PLoS One. 2017 Jun 7;12(6):e0178550. doi: 10.1371/journal.pone.0178550 (PMC5462386; doi:10.1371/journal.pone.0178550)
Supplement: S2 File — Table A. Payre, type A and type B flakes. Table B. Payre, type of platform of type A and B flakes. Table C. Bau de l’Aubesier, type A and type B flakes. Table D. Bau de l’Aubesier, type of platform of type A and type B flakes. Table E. Payre, comparison of the flake techno-types with the incidence of retouch for each category. Numbers in brackets indicate the number of retouched pieces for each category. The % ret column indicates the percentage of retouched pieces for each category. Table F. Bau de l’Aubesier, comparison of the flake techno-types with the incidence of retouch for each category. Numbers in brackets indicate the number of retouched pieces for each category. The % ret column indicates the percentage of retouched pieces for each category. (DOCX) [file pone.0178550.s002.docx]

**Technological variability during the MIS 9-7 in Western Europe. Reduction systems and predetermined products at the Bau de l’Aubesier and Payre (South-East France).**

**Supporting Information**

**Supplementary File S2.** Tables.

This PDF file includes: Tables A-F.

Table A. Payre, type A and type B flakes.

| **Levels** | **Gb** | | **Ga** | | **Fd** | | **Fc** | | **Fb** | | **Fa** | |
| --- | --- | --- | --- | --- | --- | --- | --- | --- | --- | --- | --- | --- |
|  | N | % | N | % | N | % | N | % | N | % | N | % |
| A1 Centripetal flakes with secant dorsal scars | 24 | 45.3 | 168 | 35.5 | 13 | 28.3 | 7 | 38.9 | 7 | 53.8 | 56 | 45.5 |
| A2 Debordant flakes with secant dorsal scars | 12 | 22.6 | 77 | 16.3 | 7 | 15.2 | 2 | 11.1 | 1 | 7.7 | 32 | 26.0 |
| **Subtotal (A type)** | **36** | **67.9** | **245** | **51.8** | **20** | **43.5** | **9** | **50.0** | **8** | **61.5** | **88** | **71.5** |
| B1 Centripetal flakes with parallel dorsal scars | 13 | 24.5 | 169 | 35.7 | 20 | 43.5 | 7 | 38.9 | 5 | 38.5 | 27 | 22.0 |
| B2 Debordant flakes with parallel dorsal scars | 4 | 7.5 | 59 | 12.5 | 6 | 13.0 | 2 | 11.1 | - | 0 | 8 | 6.5 |
| **Subtotal (B type)** | **17** | **32.1** | **228** | **48.2** | **26** | **56.5** | **9** | **50.0** | **5** | **38.5** | **35** | **28.5** |
| Total | 53 | 100 | 473 | 100 | 46 | 100 | 18 | 100 | 13 | 100 | 123 | 100 |

Table B. Payre, type of platform of type A and B flakes.

| **Type of platform** | **Gb** | | **Ga** | | **Fd** | | **Fc** | | **Fb** | | **Fa** | |
| --- | --- | --- | --- | --- | --- | --- | --- | --- | --- | --- | --- | --- |
|  | N | *%* | N | *%* | N | *%* | N | *%* | N | *%* | N | *%* |
| (A1) Inclined | 16 | *30.2* | 102 | *21.6* | 13 | *28.3* | 6 | *33.3* | 7 | *53.8* | 56 | *45.5* |
| (A1) Rectilinear | 8 | *15.1* | 35 | *7.4* | - | *0* | - | *0* | - | *0* | - | *0* |
| (A1) Punctiform/Linear | - | *0* | 31 | *6.6* | - | *0* | 1 | *5.6* | - | *0* | - | *0* |
| (A2) Secant | 9 | *17.0* | 44 | *9.3* | 6 | *13.0* | 2 | *11.1* | 1 | *7.7* | 28 | *22.8* |
| (A2) Rectilinear | 1 | *1.9* | 12 | *2.5* | - | *0* | - | *0* | - | *0* | 2 | *1.6* |
| (A2) Punctiform/Linear | 2 | *3.8* | 21 | *4.4* | 1 | *2.2* | - | *0* | - | *0* | 2 | *1.6* |
| (B1) Secant | 4 | *7.5* | 70 | *14.8* | 5 | *10.9* | 2 | *11.1* | - | *0* | 5 | *4.1* |
| (B1) Rectilinear | 9 | *17.0* | 59 | *12.5* | 13 | *28.3* | 5 | *27.8* | 5 | *38.5* | 19 | *15.4* |
| (B1) Punctiform/Linear | - | *0* | 40 | *8.5* | 2 | *4.3* | - | *0* | - | *0* | 3 | *2.4* |
| (B2) Secant | 4 | *7.5* | 17 | *3.6* | 2 | *4.3* | 1 | *5.6* | - | *0* | 6 | *4.9* |
| (B2) Rectilinear | - | *0* | 30 | *6.3* | 4 | *8.7* | 1 | *5.6* | - | *0* | 2 | *1.6* |
| (B2) Punctiform/Linear | - | *0* | 12 | *2.5* | - | *0* | - | *0* | - | *0* | - | *0* |
| Total | 53 | *100* | 473 | *100* | 46 | *100* | 18 | *100* | 13 | *100* | 123 | *100* |

Table C. Bau de l’Aubesier, type A and type B flakes

| **Levels** | **K2** | | **K1-K** | | **J4** | | **J3** | | **J2** | | **J1-J** | |
| --- | --- | --- | --- | --- | --- | --- | --- | --- | --- | --- | --- | --- |
|  | N | % | N | % | N | % | N | % | N | % | N | % |
| A1 Centripetal flakes with secant dorsal scars | 5 | 27.8 | 8 | 38.1 | 6 | 11.8 | 2 | 15.4 | 1 | 100 | - | 0 |
| A2 Debordant flakes with secant dorsal scars | 3 | 16.7 | 1 | 4.8 | 7 | 13.7 | - | 0 | - | 0 | 2 | 16.7 |
| B1 Centripetal flakes with parallel dorsal scars | 8 | 44.4 | 8 | 38.1 | 33 | 64.7 | 11 | 84.6 | - | 0 | 7 | 58.3 |
| B2 Debordant flakes with parallel dorsal scars | 2 | 11.1 | 4 | 19.0 | 5 | 9.8 | - | 0 | - | 0 | 3 | 25.0 |
| Total | 18 | 100 | 21 | 100 | 51 | 100 | 13 | 100 | 1 | 100 | 12 | 100 |

Table D. Bau de l’Aubesier, type of platform of type A and type B flakes.

| **Type of platform** | **K2** | | **K1-K** | | **J4** | | **J3** | | **J2** | | **J1-J** | |
| --- | --- | --- | --- | --- | --- | --- | --- | --- | --- | --- | --- | --- |
|  | N | % | N | % | N | % | N | % | N | % | N | % |
| (A1) Secant | 1 | 5,6 | 8 | 38,1 | 2 | 3,9 | 2 | 15,4 | 1 | 100 | - | 0 |
| (A1) Rectilinear | 3 | 16,7 | - | 0 | 2 | 3,9 | - | 0 | - | 0 | - | 0 |
| (A1) Punctiform/Linear | 1 | 5,6 | - | 0 | 2 | 3,9 | - | 0 | - | 0 | - | 0 |
| (A2) Secant | 1 | 5,6 | - | 0 | 7 | 13,7 | - | 0 | - | 0 | - | 0 |
| (A2) Rectilinear | - | 0 | 1 | 4,8 | - | 0 | - | 0 | - | 0 | - | 0 |
| (A2) Punctiform/Linear | 2 | 11,1 | - | 0 | - | 0 | - | 0 | - | 0 | - | 0 |
| (B1) Secant | 1 | 5,6 | 1 | 4,8 | - | 0 | - | 0 | - | 0 | - | 0 |
| (B1) Rectilinear | - | 0 | - | 0,0 | 18 | 35,3 | 5 | 38,5 | - | 0 | 3 | 25,0 |
| (B1) Punctiform/Linear | 7 | 38,9 | 7 | 33,3 | 15 | 29,4 | 6 | 46,2 | - | 0 | 4 | 33,3 |
| (B2) Secant | - | 0 | 1 | 4,8 | - | 0 | - | 0 | - | 0 | - | 0 |
| (B2) Rectilinear | - | 0 | 2 | 9,5 | 3 | 5,9 | - | 0 | - | 0 | 3 | 25,0 |
| (B2) Punctiform/Linear | 2 | 11,1 | 1 | 4,8 | 2 | 3,9 | - | 0 | - | 0 | 2 | 16,7 |
| Total | 18 | 100 | 21 | 100 | 51 | 100 | 13 | 100 | 1 | 100 | 12 | 100 |

Table E. Payre, comparison of the flake techno-types with the incidence of retouch for each category. Numbers in brackets indicate the number of retouched pieces for each category. The % ret column indicates the percentage of retouched pieces for each category.

| **Levels** | **Gb** | | **Ga** | | **Fd** | | **Fc** | | **Fb** | | **Fa** | |
| --- | --- | --- | --- | --- | --- | --- | --- | --- | --- | --- | --- | --- |
|  | N  tot(ret) | *% ret* | N  tot(ret) | *% ret* | N  tot(ret) | *% ret* | N  tot(ret) | *% ret* | N  tot(ret) | *% ret* | N  tot(ret) | *% ret* |
| Centripetal flakes | 37 (5) | *13.5* | 337 (83) | *24.6* | 33 (4) | *12.2* | 14 (4) | *28.5* | 12(2) | *16.6* | 83(22) | *26.5* |
| Debordant flakes (chordal) | 16 (5) | *31.2* | 135 (48) | *35.5* | 12 (1) | *8.3* | 3 | *0* | - | *0* | 37 (4) | *10.8* |
| Pseudolevallois | - | *0* | 1 | *0* | 1 | *0* | 1 | *0* | 1 | *0* | 3 | *0* |
| Unipolar flakes | 10 (1) | *10.0* | 24 (3) | *12.5* | 13 | *0* | 3 (1) | *33.3* | 3 | *0* | 26 (8) | *30.7* |
| Debordant unipolar flakes | 2 | *0* | 5 (1) | *20.0* | 4 | *0* | - | *0* | - | *0* | 1 (1) | *100* |
| Bipolar flakes | 1 | *0* | 2 | *0* | - | *0* | 2 | *0* | - | *0* | - | *0* |
| Debordant bipolar flakes | - | *0* | 1 | *0* | - | *0* | 2 | *0* | - | *0* | - | *0* |
| Orthogonal flakes | 1 | *0* | 5 (3) | *60.0* | - | *0* | 2 | *0* | - | *0* | - | *0* |
| Convergent/sub-convergent flakes | 2 (1) | *50.0* | 10 | *0* | - | *0* | - | *0* | - | *0* | - | *0* |
| Bladelets | - | *0* | - | *0* | 3 | *0* | - | *0* | - | *0* | - | *0* |
| Blades | - | *0* | - | *0* | 7 | *0* | - | *0* | - | *0* | - | *0* |
| Kombewa | 3 | *0* | 27 (5) | *18.5* | 1 | *0* | 1 (1) | *100* | 1 | *0* | 19 (1) | *5.2* |
| Kombewa debordant | 1 (1) | *100* | 4 (2) | *50.0* | - | *0* | - | *0* | - | *0* | 3 | *0* |
| Wide flakes | 1 | *0* | 22 (7) | *31.8* | 17 (1) | *5.8* | 6 (3) | *50.0* | 1 | *100* | 31 (9) | *29.0* |
| Striking platform flakes | 2 (1) | *50.0* | 1 | *0* | - | *0* | 7 | *0* | - | *0* | 2 | *0* |
| Shaping/retouching flakes | 4 | *0* | 43 | *0* | 1 | *0* | 3 | *0* | - | *0* | 10 | *0* |
| Rejuvenation flakes | 1 | *0* | 21 (14) | *66.6* | - | *0* | - | *0* | - | *0* | - | *0* |
| Crested flakes | 1 | *0* | - | *0* | 4 | *0* | - | *0* | 1 | *0* | 1 | *0* |
| **Total** | 82 (18) | *19.2* | 638 (166) | *26* | 96 (6) | *6.2* | 44 (9) | *20.4* | 19(2) | *15.5* | 216 (45) | *20,8* |

Table F. Bau de l’Aubesier, comparison of the flake techno-types with the incidence of retouch for each category. Numbers in brackets indicate the number of retouched pieces for each category. The % ret column indicates the percentage of retouched pieces for each category.

| **Levels** | **K2** | | **K1-K** | | **J4** | | **J3** | | **J2** | | **J1-J** | |
| --- | --- | --- | --- | --- | --- | --- | --- | --- | --- | --- | --- | --- |
|  | **num** | ***%*** | **num** | ***%*** | **num** | ***%*** | **num** | ***%*** | **num** | ***%*** | **num** | ***%*** |
| Flakes (Cortex >50%) | 5 | *0* | 4 | *0* | 12(1) | *8.3* | 3 | *0* | 2 | *0* | 10 | *0* |
| Flakes (Cortex<50%) | 11 | *0* | - | *0* | 20(1) | *5.0* | 4 | *0* | - | *0* | 40 | *0* |
| Centripetal flakes | 13(2) | *15.4* | 16(1) | *6.2* | 39(11) | *28.2* | 13 | *0* | 1 | *0* | 7(2) | *28.6* |
| Debordant flakes (chordal) | 5(2) | *28.6* | 5(1) | *20.0* | 12 | *0* | - | *0* | - | *0* | 5 | *0* |
| Unipolar flakes | 10(1) | *10.0* | 22(4) | *18.2* | 30(4) | *13.3* | 6 | *0* | 2 | *0* | 13(2) | *15.3* |
| Debordant unipolar flakes | 3(1) | *33.3* | 5(2) | *40.0* | 4 |  | - | *0* | - | *0* | 1(1) | *100* |
| Bipolar flakes | 4(2) | *50.0* | 6(4) | *66.7* | 2(1) | *50.0* | - | *0* | - | *0* | - | *0* |
| Debordant bipolar flakes | 1(1) | *100* | - | *0* | 1 | *0* | - | *0* | - | *0* | - | *0* |
| Orthogonal flakes | - | *0* | - | *0* | 1 | *0* | - | *0* | - | *0* | - | *0* |
| Debordant Orthogonal flakes | - | *0* | - | *0* | 1 | *0* | - | *0* | 1 | *0* | - | *0* |
| Convergent/sub-convergent flakes | 4(2) | *50.0* | 5(1) | *20.0* | 28(6) | *21.4* | 2 | *0* | - | *0* | 1 | *0* |
| Bladelet | 1 | *0* | - | *0* | - | *0* | - | *0* | - | *0* | - | *0* |
| Blades | 19(4) | *21.1* | 19(3) | *15.8* | 14(5) | *35.7* | 1 | *0* | 1 | *0* | 3(1) | *33.3* |
| Crested blades | 2 | *0* | 1 | *0* | - | *0* | - | *0* | - | *0* | 1 | *0* |
| Kombewa | - | *0* | 3 | *0* | 1 | *0* | - | *0* | - | *0* | - | *0* |
| Macro-outils | 2 (2) | *100* | 10(10) | *100* | 1(1) | *100* | - | *0* | - | *0* | 2(2) | *100* |
| Striking platform flakes | 3 | *0* | 5 | *0* | 6 | *0* | 1 | *0* | - | *0* | - | *0* |
| Shaping/retouching flakes | - | *0* | 1 | *0* | 3 | *0* | - | *0* | - | *0* | - | *0* |
| Rejuvenation flakes | - | *0* | 2(1) | *50.0* | 2(1) | *50.0* | - | *0* | - | *0* | - | *0* |
| Burin de Siret | 1(1) | *100* | 3 (2) | *66.6* | 8 | *0* | - | *0* | 1 | *0* | - | *0* |
| **Total** | 84 (18) | *21,4* | 107 (29) | *27,10* | 185 (31) | *16,7* | 30 | *0* | 8 | *0* | 83(8) | *9,6* |
