# Supplementary material for: Technological variability during the Early Middle Palaeolithic in Western Europe. Reduction systems and predetermined products at the Bau de l'Aubesier and Payre (South-East France)
Source: PLoS One. 2017 Jun 7;12(6):e0178550. doi: 10.1371/journal.pone.0178550 (PMC5462386; doi:10.1371/journal.pone.0178550)
Supplement: S3 File — Data of European sites from MIS 9 to 7. Payre and Bau de l’Aubesier are in bold. Certain technological identification (X). Uncertain technological identification (X?). (DOCX) [file pone.0178550.s003.docx]

**Technological variability during the MIS 9-7 in Western Europe. Reduction systems and predetermined products at the Bau de l’Aubesier and Payre (South-East France).**

**Supporting Information**

**Supplementary File S3**

**Discussion and conclusion**

This PDF file includes:

Table G

References

Table G. Data of European sites from MIS 9 to 7. Payre and Bau de l’Aubesier are in bold. Certain technological identification (X). Uncertain technological identification (X?).

| **Nation** | **Site** | **Level/Layer** | **MIS** | **Dating** | **Relative chronology** | **Levallois** | **Discoid** | **SSDA** | **Trifacial** | **Blade** | **Bladelets** | **Others** | **Handaxe** | **Pebble tools** | **Quina** | **Bibliography** |
| --- | --- | --- | --- | --- | --- | --- | --- | --- | --- | --- | --- | --- | --- | --- | --- | --- |
| Bailiwick of Jersey | La Cotte de St. Brelade | D | 7 | 238 ± 35 ka (TL) | Biostratigraphy, Biochronology | - | ✕ | ✕ | - | - | - | - | - | - | - | Calllow & Cornford 1986; Soriano 2000 |
| Bailiwick of Jersey | La Cotte de St. Brelade | C | 7 | 238 ± 35 ka (TL) | Biostratigraphy, Biochronology | ✕ | ✕ | ✕ | - | - | - | - | - | - | - | Calllow & Cornford 1986; Soriano 2000 |
| Bailiwick of Jersey | La Cotte de St. Brelade | B | 7 | - | Biostratigraphy, Biochronology | ✕ | ✕ | ✕ | - | - | - | - | - | - | - | Calllow & Cornford 1986; Soriano 2000 |
| Bailiwick of Jersey | La Cotte de St. Brelade | A | 7 | - | Biostratigraphy, Biochronology | ✕ | ✕ | ✕ | - | - | - | - | ✕ | - | - | Calllow & Cornford 1986; Soriano 2000 |
| Belgium | Mesvin IV | - | 8 | 300 to 250 ka (U-Th), 297 ± 12 ka (U-Th) | Lithostratigraphy, Biochronology, Terrace system | ✕ | ✕ | - | - | - | - | ✕ | ✕ | - | - | Cahen & Haesaerts 1981; Cahen et al. 1984; Soriano 2000 |
| Belgium | Rissori | 2b | 7 | - | Lithostratigraphy, Biochronology | ✕ | - | - | - | ✕ | - | - | - | - | - | Adam 1991, 2002; Adam & Truffeau 1973 |
| France | Abbeville | - | 7 | - | Lithostratigraphy, Biochronology, Terrace system | ✕ | - | - | - | - | - | - | - | - | - | Loch et al. 2013 |
| France | Barbas I | C4 | 7 | 239 ± 44 ka (TL) | - | - | - | - | ✕ | - | - | - | ✕ | - | - | Boeda 1991, 2001; Valladas et al 1999 |
| **France** | **Bau de l'Aubesier** | **J** | **7** | **190 to 220 ka (ESR)** | **Lithostratigraphy, Biochronology** | **✕** | **-** | **✕** | **-** | **✕** | **-** | **✕** | **-** | **-** | **-** | **Blackwell et al. 2001; Wilson & Brown 2014; Carmignani 2017** |
| **France** | **Bau de l'Aubesier** | **K** | **7** | **-** | **Lithostratigraphy, Biochronology** | **-** | **✕** | **-** | **-** | **✕** | **-** | **✕** | **-** | **-** | **-** | **Wilson & Brown 2014; Carmignani 2017** |
| France | Baume Bonne | Phase 2 | 8-7 | 200 ka (U-Th) | - | ✕ | ✕ | ✕ | - | - | - | - | ✕ | ✕ | - | Gagnepain & Gailllard 2005; Gagnepain et al. 2004 |
| France | Biache-Saint-Vaast | H | 7 | 258 ± 26 ka (ESR); 245 ± 28 ka (TL) | Lithostratigraphy, Biochronology, Terrace system | ✕ | - | - | - | - | - | ✕ | - | - | - | Bahain 2007; Bahain et al. 2015; Hérisson 2012; Amelot-Van der Heijden 1991 |
| France | Biache-Saint-Vaast | IIA | 7 | 230 ± 24 ka (ESR); 229 ± 27 ka (ESR); 219 ± 30 ka (ESR) | Lithostratigraphy, Biochronology, Terrace system | ✕ | - | - | - | ✕ | - | ✕ | - | - | - | Bahain 2007; Bahain et al. 2015; Herisson 2012 |
| France | Biache-Saint-Vaast | IIbase | 7 | 190 ± 17 ka (ESR); 222 ± 27 ka (ESR) | Lithostratigraphy, Biochronology, Terrace system | ✕ | - | - | - | - | - | ✕ | - | - | - | Bahain 2007; Bahain et al. 2015; Herisson 2012 |
| France | Biache-Saint-Vaast | E | 7 | - | Lithostratigraphy, Biochronology, Terrace system | ✕ | - | - | - | - | - | ✕ | - | - | - | Amelot-Van der Heijden 1989; 1991; Auguste 1994, 1995 |
| France | Biache-Saint-Vaast | DO | 7 | 139 ± 27 ka (ESR); 138 ± 28 ka (ESR) | Lithostratigraphy, Biochronology, Terrace system | ✕ | - | - | - | - | - | ✕ | - | - | - | Bahain 2007; Bahain et al. 2015; Amelot-Van der Heijden 1989; 1991 |
| France | Cagny L'Epinette | I | 9 | 318 ± 48 ka (US-ESR); 289 ± 43 ka (US-ESR); 291 ± 44 ka (US-ESR) | Lithostratigraphy, Biochronology, Palinology, Terrace system | - | - | ✕ | - | - | - | - | ✕ | - | - | Bahain et al 2007; Bahain et al. 2001; Laurent 1993; Antoine & Truffeau 1993; Tuffreau et al. 1995; Lamotte 1999 |
| France | Cantalouette 1 | V | 7 | 222 ± 20 ka (TL) | - | ✕ | - | - | - | - | - | - | ✕ | ✕ | - | Brenet et al. 2008; Vieillevigne et al. 1999; Brenet 2011, 2013 |
| France | Combe Brune 2 | X | 7 | 195 ± 16 ka (TL) | - | ✕ | ✕ | - | ✕ | ✕ | - | ✕ | ✕ | ✕ | - | Brenet et al. 2008; Brenet 2011, 2013; Frouin et al. 2014; |
| France | Combe Brune 2 | VIII | 8-7 | 220 to 195 ka (TL, OSL); 208 ± 8 ka (U-Th) | - | ✕ | ✕ | ✕ | ✕ | ✕ | - | - | ✕ | - | - | Brenet et al. 2008; Brenet 2011, 2013; Frouin et al. 2014; |
| France | Etricourt-Manancourt | HUZ | 9 | - | Lithostratigraphy | - | - | ✕ | - | - | - | - | ✕ | - | - | Hérisson & Goval 2013; Hérisson et al 2015; Hérisson et al 2016 |
| France | Etricourt-Manancourt | HUD | 9-8 | 274 ± 32 ka (TL); 294 ± 25 ka (TL); 288 ± 26 ka (TL) | Lithostratigraphy | ✕ | - | ✕ | - | - | - | ✕ | ✕ | - | - | Hérisson & Goval 2013; Hérisson et al 2015; Hérisson et al 2016 |
| France | La Micoque | L2-3 | 9-8 | 332 to 291 ka (ESR) | - | - | - | ✕ | ✕ | - | - | ✕ | - | - | - | Falguères et al. 1997; Guibert et al. 2008; Delpech et al. 1995 |
| France | Le Pucheuil | A to C | 8-7 | - | Lithostratigraphy | ✕ | - | - | - | ✕ | - | - | - | - | - | Delagnes 1993, Delagnes & Ropars dir. 1996; Lazuén & Delagnes 2014 |
| France | Les Bosses | - | 8 | 250 to 300 ka (TL); 291 ± 31 ka (TL) | - | ✕ | ✕ | - | - | - | - | - | ✕ | ✕ | - | Jarry et al. 2007; Jerry et al. 2004; Jarry 2010 |
| France | Orgnac | 6 | 9 | 362 ± 51 ka (ESR) | Biostratigraphy | ✕ | ✕ | ✕ | - | - | - | - | ✕ | ✕ | - | Bahain et al. 2012; Moncel et al. 2005, 2011, 2012; Moncel 1995 |
| France | Orgnac | 5 | 9 | 111 ± 161 ka (ESR); 347 ± 65 ka (ESR); 346 ± 37 ka (ESR) | Biostratigraphy | ✕ | ✕ | ✕ | - | - | - | - | ✕ | ✕ | - | Bahain et al. 2012Moncel et al. 2005, 2011, 2012; Moncel 1995 |
| France | Orgnac | 4a | 9 | - | Biostratigraphy | ✕ | ✕ | ✕ | - | - | - | - | ✕ | ✕ | - | Moncel et al. 2005, 2011, 2012; Moncel 1995 |
| France | Orgnac | 3 | 9-8 | - | Biostratigraphy | ✕ | ✕ | ✕ | - | - | - | - | ✕ | ✕ | - | Moncel et al. 2005, 2011, 2012; Moncel 1995 |
| France | Orgnac | 2 | 9-8 | - | Biostratigraphy | ✕ | ✕ | ✕ | - | - | - | - | ✕ | ✕ | - | Moncel et al. 2005, 2011, 2012; Moncel 1995 |
| France | Orgnac | 1 | 9-8 | 242 to 360 ka (ESR) | Biostratigraphy | ✕ | ✕ | ✕ | - | - | - | - | ✕ | ✕ | - | Moncel et al. 2005, 2011, 2012; Moncel 1995; Massaoudi 1995 |
| **France** | **Payre** | **F** | **8-7** | **169 ± 13 ka (ESR/U-Th); 232 ± 15 ka (TL)** | **Lithostratigraphy, Biochronology** | **-** | **✕** | **✕** | **-** | **-** | **✕** | **✕** | **✕** | **✕** | **✕** | **Valladas et al. 2008; Bahain 2009; Carmignani 2017; Moncel & Patou-Mathis 2008; Moncel 1995; Descalux et al. 2008** |
| **France** | **Payre** | **G** | **8-7** | **235 ± 18 ka (ESR/U-Th); 231 ± 27 ka (TL)** | **Lithostratigraphy, Biochronology** | **-** | **✕** | **✕** | **✕** | **-** | **-** | **✕** | **✕** | **✕** | **✕** | **Valladas et al. 2008; Bahain 2009; Carmignani 2017; Moncel & Patou-Mathis 2008; Moncel 1995; Descalux et al. 2008** |
| France | Pech de l'Aze II | c9 | 7 | 174 ± 22 ka (ESR); 131 ± (17 ka (ESR) | - | - | - | - | ✕ | - | - | - | - | ✕ | - | Grun & Stringer 1991; Delpech et al. 1995; Boeda 1991; Bordes 1984 |
| France | Pech de l'Aze II | c8 | 7 | 152 ± 16 ka (ESR); 194 ± 19 ka (ESR) | - | - | - | - | ✕ | - | - | - | - | ✕ | - | Grun & Stringer 1991; Delpech et al. 1995; Boeda 1991; Bordes 1984 |
| France | Pech de l'Aze II | c7 | 7 | 156 ± 15 ka (ESR); 193 ± 19 ka (ESR) | - | - | - | - | ✕ | - | - | - | - | ✕ | - | Grun & Stringer 1991; Delpech et al. 1995; Boeda 1991; Bordes 1984 |
| France | Petit-Bost | 2 | 9-8 | 248 ± 31 ka (TL); 312 ± 23 ka (TL); 338 ± 43 ka (TL) | - | ✕ | ✕ | - | - | - | - | ✕ | ✕ | - | ✕ | Guilbert et al. 2006; Bourguignon et al 2008; Lahaye 2005 |
| France | Ranville | A | 7 | 205 to 235 ka (U-Th) | - | - | - | - | - | - | - | ✕ | - | ✕ | - | Clique dir. 2008 |
| France | Ranville | B | 7 | 205 to 235 ka (U-Th) | - | - | - | - | - | - | - | ✕ | ✕ | ✕ | - | Clique dir. 2008 |
| France | Saint-Valery-sur-Sommes | SO | 8-7 | - | Lithostratigraphy | - | - | - | - | ✕ | - | - | - | - | - | De Heinzelin & Haesaerts 1983 |
| France | Soucy | 5-II | 9 | 356 ± 53 ka (U-Th); 399 ± 60 ka (U-Th) | Lithostratigraphy, Biochronology, Terrace system | - | ✕ | ✕ | - | - | - | - | ✕ | - | - | Lhomme et al 2000a, 2004; Chaussé 2003; Lhomme 2007 |
| France | Soucy | 5-I | 9 | 323 ± 24 ka (U-Th) | Lithostratigraphy, Biochronology, Terrace system | - | ✕ | ✕ | - | - | - | - | ✕ | - | - | Lhomme & Connet 2001; Lhomme et al. 2004; Chaussé 2003; Lhomme 2007 |
| France | Soucy | 3-P | 9 | 361 ± 30 ka (U-Th) | Lithostratigraphy, Biochronology, Terrace system | - | - | - | - | - | - | - | ✕ | - | - | Lhomme et al 2000b, 2004; Chaussé 2003; Lhomme 2007; Nicoud 2011 |
| France | Soucy | 1 | 9 | - | Lithostratigraphy, Biochronology, Terrace system | - | - | - | - | - | - | - | ✕ | - | - | Lhomme et al 1998a, 1998b, 2000b, 2004; Chaussé 2003; Lhomme 2007 |
| France | Soucy | 5-0 | 9 | 362 ± 27 ka (U-Th) | Lithostratigraphy, Biochronology, Terrace system | - | - | - | - | - | - | - | ✕ | - | - | Lhomme et al 1998a, 1998b, 2000b, 2004; Chaussé 2003; Lhomme 2007 |
| France | Soucy | 3-S | 9 | - | Lithostratigraphy, Biochronology, Terrace system | - | - | - | - | - | - | - | ✕ | - | - | Lhomme et al. 2000a, 2004; Chaussé 2003; Lhomme 2007 |
| France | Soucy | 2 | 9 | - | Lithostratigraphy, Biochronology, Terrace system | - | - | ✕ | - | - | - | - | - | - | - | Lhomme et al. 2000a, 2004; Chaussé 2003; Lhomme 2007 |
| France | Therdonne | N3 | 7 | 178 ± 11 ka (TL) | Lithostratigraphy | ✕ | ✕ | - | - | ✕ | - | - | - | - | - | Loch et al. 2010 |
| France | Tourville-la-Rivière | D1 | 7 | - | Lithostratigraphy, Biochronology, Terrace system | ✕ | - | - | - | ✕ | - | ✕ | - | - | - | Vallin 1991, Guilbaud & Carpentier 1995 |
| France | Vaufrey | IX | 7 | 208 ± 8 ka (U-Th) | - | ✕ | - | - | - | - | - | - | - | ✕ | - | Geneste 1985, 1988; Hernadez et al. 2014; Rigaud dir. 1988; |
| France | Vaufrey | X | 8-7 | 246 ka (U-Th) | - | - | ✕ | - | - | - | - | - | ✕ | ✕ | - | Geneste 1985, 1988; Hernadez et al. 2014; Rigaud dir. 1988; |
| Germany | Ariendorf | 1 | 8 | - | Lithostratigraphy | ✕ | - | - | - | - | - | - | - | - | - | Bosinski et al. 1983; Turner et al. 1997; Richter 2011 |
| Germany | Ehringsdorf | LT | 7 | 204 ka medium age (ESR) | Lithostratigraphy, Biochronology | - | - | - | - | - | - | ✕ | ✕ | - | - | Heinrich 1981, Schafer 1993, Schuler 2004 |
| Germany | Ehringsdorf | UT | 7 | 204 ka medium age (ESR) | Lithostratigraphy, Biochronology | - | - | - | - | - | - | ✕ | ✕ | - | - | Heinrich 1981, Schafer 1993, Schuler 2004 |
| Germany | Rheindahlen | B3 | 7 | 142 to 194 ka (TL) | Lithostratigraphy | ✕ | - | - | - | - | - | - | - | - | - | Schmitz & Thissen 1998, Thissen 2006; Frechen et al. 1992; Zoller et al. 1988; Ikinger 2002 |
| Germany | Rheindahlen | B2 | 7 | 142 to 194 ka (TL) | Lithostratigraphy | - | - | - | - | - | - | - | ✕ | - | - | Schmitz & Thissen 1998, Thissen 2006; Frechen et al. 1992; Zoller et al. 1988; Ikinger 2002 |
| Germany | Rheindahlen | B1 | 7 | 142 to 194 ka (TL) | Lithostratigraphy | - | - | - | - | ✕ | - | - | - | - | - | Schmitz & Thissen 1998, Thissen 2006; Frechen et al. 1992; Zoller et al. 1988; Ikinger 2002 |
| Germany | Shoningen | 12-B, 12-II | 9-8 | - | Lithostratigraphy | - | - | - | - | - | - | ✕ | - | - | - | Thieme 2007; Serangeli et al 2012 |
| Germany | Shoningen | 13-II-4 | 9-8 | - | Lithostratigraphy | - | - | - | - | - | - | ✕ | - | - | - | Serangeli & Conard 2015 |
| Italy | Cave dall'Olio | - | 9 | - | Lithostratigraphy | ✕ | ✕ | ✕ | - | ✕ | - | - | - | - | - | Fontana et al 2009; Fontana et al. 2013 |
| UK | Baker's Hole "Northflleet" | - | 8 | - | Lithostratigraphy, Biochronology, Terrace system | ✕ | - | ✕ | - | ✕(?) | - | - | ✕ | - | - | Wenban-Smith 1995, 1996; Scott 2010, 2011 |
| UK | Crayford "Stoneham's Pit" | - | 7 | - | Biochronology, Terrace system | ✕ | ✕ | ✕ | - | ✕(?) | - | - | - | - | - | Spurrel 1880; Cook 1986 |
| UK | Harnham | Phase I | 8 | - | Lithostratigraphy | - | - | - | - | - | - | - | ✕ | - | - | Bates et al. 2014 |
| UK | Harnham | Phase II | 8 | - | Lithostratigraphy | - | - | - | - | - | - | - | - | - | - | Bates et al. 2014 |
| UK | Harnham | Phase III | 8 | 248 ± 19 ka (OSL); 255 ± 20 ka (OSL) | Biostratigraphy | - | - | - | - | - | - | - | ✕ | - | - | Bates et al. 2014 |
| UK | Harnham | Phase IV | 8 |  | Lithostratigraphy | - | - | - | - | - | - | - | ✕ | - | - | Bates et al. 2014 |
| UK | Pontnewydd | - | 7 | 200 ± 25 ka (TL); 269 ± 37 ka (TL) | Biostratigraphy | ✕ | ✕ | ✕ | - | - | - | ✕ | ✕ | - | - | Aldhouse-Green et al 2012 |
| UK | Purfleet | Botany Gravel | 9-8 | 154 ± 19 ka (OSL); 323 ± 23 ka (OSL); 292 ± 43 ka (OSL); 360 ± 62 ka (OSL) | Biochronology, Terrace system | ✕ | ✕ | ✕ | - | - | - | ✕ | - | - | - | Penkman et al. 2007; Bridgland et al. 2013; White & Ashton 2003; Scott 2011 |

**References**

Adam A (1991) Le gisement paléolithique moyen du Rissori à Masnuy-Saint-Jean (Hainaut, Belgique): premiers résultats. In: Paléolithique et Mésolithique du Nord de la France, Nouvelles recherches II (Tuffreau, A., Ed.), Publications du CERP Vol. 3, Centre d'Études et de Recherches Préhistoriques (CERP), Université des Sciences et Technologies de Lille, Villeneuve d'Ascq: 41-52.

Adam A (2002) The pseudo-Levallois points of the Mousterian site Le Rissori at Masnuy-Saint-Jean (Hainaut, Belgium). L'Anthropologie, 106(5), 695-730.

Adam A. Tuffreau A (1973) Le gisement paléolithique ancien du Rissori, à Masnuy-Saint-Jean (Hainaut, Belgique). Bulletin de la Société préhistorique française. 70(1): 293–310. doi:10.3406/bspf.1973.4377

Aldhouse-Green S, Peterson R, Walker E (2012) Neanderthals in Wales: Pontnewydd and the Elwy Valley Caves. Oxbow Books.

Ameloot-Van der Heijden N (1989) Les séries lithiques des niveaux E et de la couche D0 du gisement paléolithique moyen de Biache-Saint-Vaast (Pas-de- Calais). In: Tuffreau A (Ed.), Paléolithique et Mésolithique Du Nord de La France: Nouvelles Recherches. Villeneuve-d'Ascq: 43-50.

Ameloot-Van der Heijden N (1991) Méthodes d'acquisition et signification des industries lithiques au Paléolithique moyen: l'exemple des gisements de plein air du Nord de la France. Université des Sciences et Technologies de Lille Flandres Artois, Villeneuve-d’Ascq.

Antoine P, Tuffreau A (1993) Contexte stratigraphique, climatique et paléotopographique des occupations acheuléennes de la moyenne terrasse de la Somme. Bulletin de la Société préhistorique française (90): 243-250. http:// dx.doi.org/10.3406/bspf.1993.9589.

Auguste P (1994) Relations entre la taphocénose et l'évolution géologique d'un site préhistorique: l'apport du gisement Pléistocène Moyen de Biache-Saint-Vaast (Pas-de-Calais, France): Actions climatique et édaphique. Artefacts, 9: 29-40.

Auguste P (1995) Cadres biostratigraphique et paléoécologique du peuplement humain dans la France septentrionale durant la Pléistocène. Apports de l'étude paléontologique des grands mammifères du gisement de Biache-Saint-Vaast(Pas-de-Calais), Doctoral dissertation, Museum Nationelle d’Histoire Naturelle, Paris.

Bahain JJ (2007) La méthode de datation par résonance de spin électronique (ESR) au Muséum national d'histoire naturelle. Vingt ans de recherches méthodologiques et d'applications géochronologiques. Mémoire d'habilitation à diriger des recherches. Université Michel de Montaigne, Bordeaux 3, Bordeaux.

Bahain JJ (2009) La datation au Carbone 14. In: Depaepe P (Ed.), La France du Paléolithique. Editions La Découverte, Paris, p. 104.

Bahain JJ, Falguères C, Laurent M (2001) Datation par résonance paramagnétique électronique (RPE) de sédiments et par combinaison des méthodes RPE/U-Th de restes paléontologiques(provenant des sites paléolithiques de Cagny). Publications du CERP: 37-40.

Bahain JJ, Falguères C, Laurent M, Shao Q, Dolo JM, Garcia T, Douville E, Frank N., Monnier JL, Hallégouët B, Laforge M, Huet B, Auguste P, Liouville M, Serre F, Gagnepain J (2012) ESR and ESR/U-series dating study of several middle Palaeolithic sites of Pléneuf-Val-André (Brittany, France): Piégu, Les Vallées and Nantois. Quaternary Geochronology, 10: 424-429. http:// dx.doi.org/10.1016/j.quageo.2012.02.013.

Bahain JJ, Falguères C. Laurent M, Voinchet P, Dolo JM, Antoine P, Tuffreau A (2007) ESR chronology of the Somme River Terrace system and first human settlements in Northern France. Quaternary Geochronology (2): 356-362. <http://dx.doi.org/10.1016/j.quageo.2006.04.012>.

Bates MR, Wenban-Smith FF, Bello SM, Bridgland DR, Buck LT, Collins MJ, Keen DH, Leary J, Parfitt SA, Penkman K, Rhodes E, Ryssaert C, Whittaker JE (2014) Late persistence of the Acheulian in southern Britain in an MIS 8 interstadial: evidence from Harnham, Wiltshire. Quaternary Science Reviews 101: 159-176. http://dx.doi.org/10.1016/j.quascirev.2014.07.002.

Blackwell BAB, Skinner AR, Blickstein JIB, Lebel S, Leung HYM (2001) ESR isochron dating analyses at Bau de l’Aubesier, Provence, France: clues to U uptake in fossil teeth. Geoarchaeology, 16: 719-761.

Boëda E (1991) Approche de la variabilité des systèmes de production lithique des industries du Paléolithique inférieur et moyen: chronique d'une variabilité attendue. Technique et culture 17(18): 37-79.

Boëda E (1991) La conception trifaciale d'un nouveau mode de taille paléolithique. Les premiers Européens. In : Actes du Congrès National des Sociétés Savantes, Commission de Pré et Protohistoire, 114: 251-263.

Boëda E (2001) Détermination des unités techno-fonctionnelles de pièces bifaciales provenant de la couche acheuléenne C’3 base du site de Barbas I. In: Cliquet D (Ed.), Les industries à outils bifaciaux du Paléolithique moyen d’Europe occidentale. Actes de la Table Ronde Internationale Organisée à Caen (Basse Normadie - France) 14 et 15 Octobre, p. 51-75.

Bordes F (1984) Leçons sur le Paléolithique. In: le Paléolithique en Europe, tome II. CNRS Edition. Ed. Cahiers du Quaternaire, 7, Paris.

Bosinski G, Brunnacker K, Turner E (1983) Ein Siedlungsbefund des Frühen Mittelpaläolithikums von Ariendorf, Kr. Neuwied. Archäologisches Korrespondenzblatt Mainz, 13(2): 157-169.

Bourguignon L, Djema H, Bertran P, Lahaye C, Guibert P (2008) Le gisement Saalien de Petit-Bost (Neuvic, Dordogne) al’origine du Moustérien d’Aquitaine. Les societes du Paleolithique dans un Grand Sud-Ouest de la France: Nouveaux gisements, nouveaux resultats, nouvelles methodes. Journees SPF, Universite Bordeaux, 1: 44-55.

Brenet M (2011) Variabilité et signification des productions lithiques au Paléolithique moyen ancien: l’exemple de trois gisements de plein-air du Bergeracois (Dordogne, France) (Doctoral dissertation, Université de Bordeaux I, Bordeaux.

Brenet M (2013) Variabilité et signification des productions au Paléolithique moyen ancien. L'exemple de trois gisements de plein-air du Bergeracois (Dordogne, France). BAR International Series, n 2548, p. 355

Brenet M, Folgado M, Bertran P, Lenoble A, Guibert P, Vieillevigne EE (2008) Interprétation de la variabilité technologique de deux industries du Paléolithique moyen ancien du Bergeracois: Cantalouette 1 et Combe Brune 3 (Creysse, Dordogne). Contexte géoarcheologique et chronologique, analyse techno-économique. In: Jaubert J, Bordes JG, Ortega I (Eds.), Les Société Paléolithiques D’un Grand Sud-Ouest: Nouveaux Gisements, Nouvelles Méthodes, Nouveaux Résultats. Mémoires de La Société Préhistorique Française, XLVII. Société Préhistorique Française: 57-81.

Bridgland DR, Harding P, Allen P, Candy I, Cherry C, George W, Horne DJ, Keen DH, Penkman KEH, Preece RC, Rhodes EJ, Scaife R, Schreve DC, Schwenninger JL, Slipper I, Ward GR, White MJ, White TS, Whittaker JE (2013) An enhanced record of MIS 9 environments, geochronology and geoarchaeology: data from construction of the High Speed 1 (LondoneChannel Tunnel) rail-link and other recent investigations at Purfleet, Essex, UK. Proceedings of the Geologists' Association 124: 417-476. http://dx. doi.org/10.1016/j.pgeola.2012.03.006.

Cahen D & Haesaerts P (1980) Le site paléolithique moyen de Mesvin. Archaeologia Belgica Bruxelles, (238): 5-9.

Cahen D, Haesaerts P, Szabo BJ, Van Neer W, Wanet P (1983) An early middle palaeolithic site at Mesvin IV (Mons, Belgium). Its significance for stratigraphy and palaeontology. Bulletin-Institut royal des sciences naturelles de Belgique. Sciences de la terre, 55(5): 1-20.

Callow P, Cornford JM (eds.) (1986) La Cotte de St. Brelade (Jersey) 1961-1978. Excavations by C.B.M. Mc Burney. Geo Books, Norwich, 433 p.

Carmignani L (2017) From the flake to the blade. The technological evolution of the Middle Paleolithic blade phenomenon. Doctoral Thesis, Universitat Rovira I Virgili, Tarragona, Spain p. 264.

Chaussé C (2003) Les nappes alluviales de la basse vallée de l'Yonne, approche géométrique et chronostratigraphique et l'apport de l'étude de la Nappe de Soucy à la compréhension des occupations du Paléolithique inférieur de Soucy, Doctoral dissertation, Lille 1.

Cliquet D. (Ed.), (2008). Le site pléistocène moyen récent de Ranville (Calvados-France) dans son contexte environnemental: analyse du fonctionnement d'une aire de boucherie soutirée par un réseau karstique. ERAUL, p. 119.

Cook J (1986) A blade industry from Stoneham’s Pit, Crayford (Kent). In: The Palaeolithic of Britain and its Nearest Neighbours: Recent Trends, (Collcutt, S.N., Ed.), Department of Prehistory & Archaeology, University of Sheffield, Sheffield: 16-19.

Delagnes A (1993) Un mode de production inédit au Paléolithique moyen dans l'industrie du niveau 6e du Pucheuil (Seine-Maritime). Paléo, 5: 111-120. doi:10.3406/pal.1993.1106.

Delagnes A, Ropars A (1996) Paléolithique moyen en pays de Caux (Haute-Normandie). Le Pucheuil, Etoutteville: deux gisements de plein air en milieu loessique, Vol. 56. Éditions de la Maison des sciences de l'homme. 243 p.

Delpech F, Geneste JM, Rigaud JP, Texier JP (1995) Les industries antérieures à la dernière glaciation en Aquitaine septentrionale: chronologie, paléoenvironnements, technologie, typologie et économie de subsistance. Paléo. Supplément. 1(1) : 133-163

Desclaux E, El Hazzazi N, Vilette P, Dubar M (2008) Le contexte paléoenvironnemental des occupations humaines. L’apport de la microfaune, des restes aviaires et de la malacofaune. In: Moncel MH Editor, Le site de Payre, occupations humaines dans la vallée du Rhône à la fin du Pléistocène moyen et au début du Pléistocène supérieur. Mémoire de la Société Préhistorique Française XLVI: 91-106.

Falguéres C, Bahain JJ, Saleki H (1997) U-series and ESR dating of teeth from Acheulian and Mousterian levels at La Micoque (Dordogne, France). Journal of Archaeological Science 24: 537-545. http://dx.doi.org/10.1006/jasc.1996.0137.

Fontana F, Moncel MH, Nenzioni G, Onorevoli G, Peretto C, Combier J (2013) Widespread diffusion of technical innovations around 300,000 years ago in Europe as a reflection of anthropological and social transformations? New comparative data from the western Mediterranean sites of Orgnac (France) and Cave dall’Olio (Italy). Journal of Anthropological Archaeology 32/4: 478-498.

Fontana F, Peretto C, Nenzioni G (2009) First recognition of predetermined core reduction sequences in the Southern Po Plain area before MIS 8 at the site of Cave dall’Olio (Bologna, Italy): an “ancient series” revisited. Human Evolution. 24(1): 43-56.

Frechen M, Brückner H, Radtke U (1992) A comparison of different TL-techniques on loess samples from rheindahlen (F.R.G.). Quaternary Science Reviews 11: 109-113.

Frouin M, Lahaye C, Hernandez M, Mercier N, Guibert P, Brenet M, Folgado-Lopez M, Bertran P (2014) Chronology of the Middle Palaeolithic open-air site of Combe Brune 2 (Dordogne, France): a multi luminescence dating approach. Journal of Archaeological Science 52: 524-534. http://dx.doi.org/10.1016/ j.jas.2014.09.012.

Gagnepain J, Gaillard C (2005) La grotte de la Baume Bonne (Quinson, Alpes de Haute-Provence): synthèse chronostratigraphique et séquence culturelle d'après les fouilles récentes (1988-1997). In: Données récentes sur les modalités de peuplement et sur le cadre chronostratigraphique géologique et paléoanthropologique des industries du Paléolithique inférieur et moyen en Europe, Colloque International de Rennes, septembre 2003, p. 73-86.

Gagnepain J, Gaillard C, Notter O (2004) La composante laminaire dans les industries lithiques du Paléolithique moyen du Verdon (sud-est de la France). In: Le Paléolithique moyen. [The Middle Palaeolithic], Actes du colloque UISPP, Université de Liège, 2001, Section 5, Bar International Séries, 1239: 57-65.

Geneste JM (1985) Analyse lithique d'industries mousteriennes Perigord: une approche technologique du comportement des groupes humains au Paleo- lithique moyen. Doctoral dissertation, Universite de Bordeaux I.

Geneste JM (1988) Les industries de la grotte Vaufrey: technologie du débitage, économie et circulation de la matière première lithique. In: Rigaud JP (Ed.) La Grotte Vaufrey: Paléoenvironnement, Chronologie, Activités Humaines. Société Préhistorique Française ; p. 441-518.

Grün R, Stringer CB, (1991) Electron Spin Resonance dating and the evolution of modern humans. Archaeometry 33: 153-199. http://dx.doi.org/10.1111/j.1475- 4754.1991.tb00696.x.

Guibert P, Bechtel F, Bourguignon L, Brenet M, Couchoud I, Delagnes A, Delpech F, Detrain L, Duttine M, Folgado M (2008) Une base de donne es pour la chronologie du paléolithique moyen dans le Sud-Ouest de la France. In: Jaubert J, Bordes JG, Ortega I (Eds.) Les Sociétés Du Paléolithique Dans Un Grand Sud-Ouest: Nouveaux Gisements, Nouveaux Résultats, Nouvelles Méthodes. Mémoires de la Société préhistorique française, XLVII (Journées SPF, Université Bordeaux 1, Talence, 24-25 novembre 2006): 19-40.

Guibert P, Lahaye C, Duttine M, Bechtel F (2006) TL-dating of Mousterian open air sites of the Isle valley: Les Forêts and Petit Bost (Dordogne, France). In 34th International Symposium on Archaeometry. Centro de Estudios Borjanos: 67-72

Guilbaud M, Carpentier G (1995) Un remontage exceptionnel a Tourville-la- Rivière (Seine-Maritime). Bulletin de la Société préhistorique française 92: 289-295.

Heinrich WD (1981) Zur stratigraphischen Stellung der Wirbeltierfaunen aus den Travertinfundstätten von Weimar-Ehringsdorf und Taubach in Thüringen. Zeitschrift für Geologische Wissenschaften, 9(9): 1031-1055.

Heinzelin J, Haesaerts P (1983) Un cas de débitage laminaire au Paléolithique ancien: Croix-l’Abbé à Saint-Valery-sur-Somme. Gallia préhistoire 26(1): 189-201.

Hérisson D (2012) Etude des comportements des premiers Néandertaliens du Nord de la France : les occupations saaliennes des gisements de Biache-Saint-Vaast et de Therdonne. Thèse de doctorat. Université de Lille, Villeneuve-d'Ascq.

Hérisson D, Coutard S, Goval E, Locht JL, Antoine P, Chantreau Y, Debenham N (2016) A new key-site for the end of Lower Palaeolithic and the onset of Middle Palaeolithic at Etricourt-Manancourt (Somme, France). Quaternary International 409: 73-91. http://dx.doi.org/10.1016/ j.quaint.2016.01.055.

Hérisson D, Goval E (2013) Du Paléolithique inférieur au début du Paléolithique supérieur dans le Nord de la France: lumiére sur les premiéres découvertes du Canal Seine-Nord Europe. In: Notae Praehistoricae (33): 91-104.

Hérisson D, Goval E, Antoine P, Bahain JJ, Balescu S, Chantreau Y, Claud E, Coutard S, Debenham N, Font C, Locht JL, Pirson S, Spagna P (2015) Les occupations du Paléolithique inférieur et du Paléolithique moyen d'Etricourt-Manancourt, Canal Seine Nord Europe. Rapport de fouille, SRA de Picardie.

Hernandez M, Mercier N, Rigaud JP, Texier JP, Delpech F (2014) A revised chronology for the Grotte Vaufrey (Dordogne, France) based on TT-OSL dating of sedimentary quartz. Journal of human evolution 75: 53-63. http://dx.doi.org/ 10.1016/j.jhevol.2014.05.010.

Ikinger E. M. (2002) Zur formenkundlich-chronologischen Stellung der Rheindahlener Funde: Micoquien, Rheindahlien, MTA? In: Schirmer W (Ed.), Lösse und Böden in Rheindahlen. GeoArchaeoRhein, 5: 79- 138.

Jarry M (2010) Les groupes humains du Pléistocène moyen et supérieur en Midi toulousain: contextes, ressources et comportements entre Massif Central et Pyrénées, Doctoral dissertation, Université Toulouse le Mirail-Toulouse II.

Jarry M, Bertran P, Colonge D, Lelouvier LA, Mourre V (2004) Le gisement paléolithique moyen ancien des Bosses à Lamagdelaine (Lot, France). BAR International Series 1239: 177-186.

Jarry M, Colonge D, Lelouvier LA, Mourre V (2007) Les Bosses 1, Lamagdelaine (Lot): un gisement paléolithique moyen inférieur à l'avant-dernier interglaciaire sur la moyenne terrasse du Lot. Bulletin de la Société Préhistorique Française, Travaux 7.

Lahaye C (2005) Nouveaux apports de la thermoluminescence à la chronologie du Paléolithique dans le Sud-Ouest de la FranceEtudes en milieu hétérogène et en présence de déséquilibres radioactifs dans les séries de l'uranium, Doctoral dissertation, Bordeaux 3.

Lamotte A (1999) L'apport des remontages dans la compréhension des méthodes de débitage et de façonnage des gisements acheuléens de la Somme. Bulletin de la Société préhistorique française 96: 117-131. http://dx.doi.org/10.3406/ bspf.1999.10936.

Laurent M (1993) Datation par Résonance de Spin Electronique(ESR) de quartz de formations quaternaires. Comparaison avec le paléomagnétisme, Doctoral dissertation, Museum Nationelle d’Histoire Naturelle, Paris.

Lazuén T, Delagnes A (2014) Lithic Tool Management in the Early Middle Paleolithic: an Integrated Techno-Functional Approach Applied to Le Pucheuil-Type Production (Le Pucheuil, Northwestern France), Journal of Archaeological Science, 52, p. 337 – 353.

Lhomme V (2007) Tools, space and behaviour in the Lower Palaeolithic: discoveries at Soucy in the Paris basin. Antiquity, 81(313): 536-554.

Lhomme V, Bemilli C, Beyries S, Christensen M, Connet N. (1998) Soucy 1 (Yonne): interprétations et réflexions sur un site du Pléistocène moyen en contexte alluvial. JP Brugal, L. Meignen, M. Patou-Mathis (Eds.) Économie préhistorique: Les comportements de subsistance au Paléolithique. XVIIIe Rencontres Internationales d’Archéologie et d’Histoire d’Antibes, Association pour la Promotion et la Diffusion des Connaissances Archéologiques, Sophia Antipolis, France, p. 260-271.

Lhomme V, Bémilli C, Chaussé C, Connet N, Argant A, Bahain J, Voinchet P (2000b) Soucy 3, bilan intermédiaire de l'étude Service Régional de l'Archéologie de Bourgogne, Dijon.

Lhomme V, Connet N (2001) Observations sur les pièces bifaciales et les chaînes opératoires de façonnage dans les sites pléistocènes moyen de Soucy (Yonne), D. Cliquet dir., Les industries à outils bifaciaux du Paléolithique moyen d'Europe occidentale. In: Actes de la table-ronde internationale organisé à Caen, 14 et 15 octobre 1999, ERAUL 98 : 43-50.

Lhomme V, Connet N, Bémilli C, Chaussé C, Beyries S, Guérin C (2000) Essai d'interprétation du site paléolithique inférieur de Soucy 1 (Yonne). Gallia préhistoire, 42(1): 1-44.

Lhomme V, Connet N, Chaussé C, Bémilli C, Bahain JJ, Voinchet P (2004) Les sites et les industries lithiques du Paléolithique inférieur, moyen et supérieur de la basse vallée de l'Yonne dans leurs contextes chronostratigraphiques. Bilan de dix ans d'activité archéologique pluridisciplinaire dans le sud-est du Bassin parisien. Bulletin de la Société préhistorique française : 701-739.

Lhomme, V., Bemilli, C, Chaussé C, Connet N, Van Kolfschoten T, Limondin-Lozouet N (1998) Le gisement paléolithique inférieur de Soucy 5 (Yonne). Revue archéologique de l'Est 49: 5-30.

Locht J-L, Antoine P, Hérisson D. Gadebois G, Debenham N (2010) Une occupation de la phase ancienne du Paléolithique moyen à Therdonne (Oise). Chronostratigraphie, production de pointes Levallois et réduction des nucleus. Gallia Préhistoire 52: 1-32.

Locht JL, Coutard S, Antoine P, Sellier N, Ducrocq T, Paris C, Guerlin O, Kiefer D, Defaux F, Deschodt L, Limondin-Lozouet N (2013) Données inédites sur le Quaternaire et le Paléolithique du nord de la France. Revue Archéologique de Picardie, 3(4): 5-70.

Massaoudi H (1995) Application des méthodes du déséquilibre des familles de l’uranium (230Th/234U) et de la résonance de spin électronique (ESR) à la datation des sites d’Orgnac 3, de Payre et de l’abri des Pêcheurs (Ardèche). Doctoral disseretation, Muséum National d’Histoire Naturelle, Paris, 155 p.

Moncel MH, Moigne AM, Combier J. (2005) Pre-Neandertal behaviour during isotopic stage 9 and the beginning of stage 8. New data concerning fauna and lithics in the different occupation levels of Orgnac 3 (Ardèche, South-East France): occupation types. Journal of Archaeological Science. 32(9):1283–1301.

Moncel MH, Moigne AM, Combier J. (2012) Towards the Middle Palaeolithic in Western Europe: The case of Orgnac 3 (southeastern France). Journal of Human Evolution. 63 (5):653–66.

Moncel MH, Moigne AM, Sam Y, Combier J (2011). The emergence of Neanderthal technical behavior: new evidence from Orgnac 3 (Level 1, MIS 8), Southeastern France. Current Anthropology 52: 37-75.

Moncel MH, Patou-Mathis M (2008) Les différentes phases d’occupations humaines à Payre et hypothèses sur les types d’occupation. In: Moncel MH (Ed) Le site de Payre, occupations humaines dans la vallée du Rhône à la fin du Pléistocène moyen et au début du Pléistocène supérieur. Mémoire de la Société Préhistorique Française XLVI: 309-315.

Moncel MH. (1995) Biface et outil-biface du Paléolithique moyen ancien : Réflexion à partir des sites d’Ardèche-Orgnac 3 et Payre. Paléo. 7(1):157–69.

Nicoud E (2011) Le phénomène acheuléen en Europe occidentale: Approche chronologique, technologie lithique et implications culturelles, Doctoral dissertation, Aix Marseille 1.

Penkman KEH, Preece RC, Keen DH, Maddy D, Schreve DC, Collins MJ (2007) Testing the aminostratigraphy of fluvial archives: the evidence from intra-crystalline proteins within freshwater shells. Quaternary Science Reviews 26: 2958-2969.

Richter J (2011) When did the Middle Paleolithic begin? In: Conard NJ, Richter Jr Editors, Neanderthal lifeways, subsistence and technology: one hundred fifty years of Neanderthal study. New York; Springer:7-14.

Rigaud JP, Aitken M, Andrieux C, Beyries S, Binford L (1988) La grotte Vaufrey à Cenac et Saint-Julien (Dordogne)-Paléoenvironnements, chronologie et activités humaines. Mémoires de la Société préhistorique française, 19.

Schäfer D (1993) Grundzüge der technologischen Entwicklung und Klassifikation vor-jungpaläolithischer Steinartefakte in Mitteleuropa. Bericht der Römisch-Germanischen Kommission, 74: 49-193.

Schmitz RW, Thissen J (1998) Vorbericht über die Grabungen 1995-1997 in der mittelpaläolithischen B1-Fundschicht der Ziegeleigrube Dreesen in Rheindahlen. Archäologisches Korrespondenzblatt, 28(4): 483-498.

Schüler T (2004) ESR dating of a new palaeolithic find layer of the travertine site of Weimar-Ehringsdorf (Central Germany). In: 18th International Senckenberg Conference, Weimar; p. 233-235.

Scott B (2010) The investigation of Baker's Hole, Northfleet, Kent, 1909-1914; the impact of collection history upon the interpretation of archaeological data. Proceedings of the Geologists' Association 121: 77-82. http://dx.doi.org/ 10.1016/j.pgeola.2010.02.006.

Scott B (2011) Becoming Neanderthals: the Earlier British Middle Palaeolithic (Oxbow. ed. Oxford).

Serangeli J, Böhner U, Hassmann H, Conard NJ (2012) Die pleistozänen Fundstellen in Schöningen–eine Einführung. In: Die chronologische Einordnung Der Paläolithischen Fundstellen von Schöningen, Römish-Germanisches Zentralmuseum, Mainz, p. 1-22.

Serangeli J, Conard NJ (2015) The behavioral and cultural stratigraphic contexts of the lithic assemblages from Schöningen. Journal of Human Evolution 89: 287-297 http:// dx.doi.org/10.1016/j.jhevol.2015.07.004.

Soriano S (2000) Outillage bifacial et outillage sur éclat au Paléolithique ancien et moyen: coexistence et interaction. Doctoral Thesis, Université Paris X-Nanterre.

Spurrell FCJ (1880) On the discovery of the place where Palaeolithic implements were made at Crayford. Quarterly Journal of the Geological Society 36: 544-548.

Thieme H (2008) Die Schöninger Speere. Mensch und Jagd vor 400 000 Jahren. Stuttgart.

Thissen J (2006) Die paläolithischen Freilandstationen von Rheindahlen im Löss zwischen Maas und Niederrhein, 59. Rheinishe Ausgrabungen. Verlag von Zabern.

Turner E, Boenick W, Frechen M, Van Kolfschoten T, Schnepp E, Sefkov E, Steensma A (1997) Ariendorf—Quaternary deposits and Palaeolithic excavations in the Karl Schneider gravel pit. Jahrbuch des Römisch-Germanisches Zentralmuseum, Mainz 44: 3-191.

Valladas H, Mercier N, Ayliffe LK, Falguères C, Bahain JJ, Dolo JM, Froget L, Joron JL, Masaoudi H, Reyss JL, Moncel MH (2008) Radiometric dates for the Middle Palaeolithic sequence of Payre (Ardèche, France). Quaternary Geochronology. 3 (4): 377–89.

Valladas H, Mercier N, Falguères C, Bahain J (1999) Contribution des méthodes nucléaires à la chronologie des cultures paléolithiques entre 300 000 et 35 000 ans BP. Gallia préhistoire. http://dx.doi.org/10.3406/galip.1999.2163.

Vallin L (1991) Un site de boucherie probable dans le Pléistocène moyen de Tourville-la-Rivière (Seine-Maritime). Cahiers du Quaternaire, 16: 241-260.

Vieillevigne E, Bourguignon L, Ortega I, Guibert P (2008) Analyse croisée des données chronologiques et des industries lithiques dans le grand sud-ouest de la France (OIS 10 a 3). PALEO. Revue d'archéologie préhistorique 20: 145-166.

Wenban-Smith FF (1995) The Ebbsfleet Valley, Northfleet (Baker's hole). In: The Quaternary of the Lower Reaches of the Thames: Field Guide. Quaternary Research Association, Durham: p 147-164.

Wenban-Smith FF (1996) The Palaeolithic Archaeology of Baker's Hole: a Case Study for Focus in Lithic Analysis. University of Southampton.

White M, Ashton N (2003). Lower Palaeolithic core technology and the origins of the Levallois method in north-western Europe. Current Anthropology 44: 598- 609. http://dx.doi.org/10.1086/377653.

Wilson L, Browne CL (2014) Change in raw material selection and subsistence behaviour through time at a Middle Palaeolithic site in southern France. Journal of Human Evolution, 75: 28-39. doi:10.1016/j.jhevol.2013.12.018

Zöller L, Stremme H, Wagner GA (1988) Thermolumineszenz-Datierung an Löss-Paläoboden-Sequenzen von Nieder, Mittel-und Oberrhein/Bundesrepublik Deutschland. Chemical Geology: Isotope Geoscience 73(1): 39-62.
